# A Phase 1b/2 Study of TP-0903 and Decitabine Targeting Mutant *TP53* and/or Complex Karyotype in Patients with Untreated Acute Myeloid Leukemia ≥Age 60 Years

**DOI:** 10.1158/2767-9764.CRC-25-0091

**Published:** 2025-07-14

**Authors:** Eric D. Eisenmann, Ronan Swords, Ying Huang, Shelley Orwick, Daelynn Buelow, Nicole Abbott, Mitch Phelps, Joshua Zeidner, Matthew C. Foster, Tara L. Lin, Maria R. Baer, Yazan F. Madanat, Tibor Kovacsovics, Robert Redner, Zeina Al-Mansour, Bhavana Bhatnagar, Mona Stefanos, Molly Martycz, Franchesca Druggan, Timothy L. Chen, Ashley O. Yocum, Uma Borate, Brian J. Druker, Amy Burd, Ross L. Levine, John C. Byrd, Sharyn D. Baker, Alice S. Mims

**Affiliations:** 1 The Ohio State University, Columbus, Ohio.; 2 Oregon Health & Science University, Portland, Oregon.; 3 University of North Carolina, Chapel Hill, North Carolina.; 4 University of Kansas, Kansas City, Kansas.; 5 University of Maryland Greenebaum Comprehensive Cancer Center, Baltimore, Maryland.; 6 University of Texas Southwestern Medical Center, Dallas, Texas.; 7 The University of Utah, Salt Lake City, Utah.; 8 University of Pittsburgh Medical Center, Pittsburgh, Pennsylvania.; 9 University of Florida, Gainesville, Florida.; 10 West Virginia University Cancer Institute, Wheeling, West Virginia.; 11 The Leukemia & Lymphoma Society, Rye Brook, New York.; 12 Memorial Sloan Kettering Cancer Center, New York City, New York.; 13 University of Cincinnati, Cincinnati, Ohio.

## Abstract

**Purpose:**

Older patients who have acute myeloid leukemia (AML) with mutant *TP53* and/or complex karyotype have a dismal prognosis and lack treatment options. Having previously demonstrated that TP-0903, a multikinase inhibitor, has compelling preclinical activity in drug-resistant AML, including *TP53*-mutated AML, we evaluated the clinical activity of TP-0903 in combination with decitabine.

**Patients and Methods:**

This was a multicenter, open-label, phase 1b/2 substudy of Beat AML Master Trial (ClinicalTrials.gov: NCT03013998). The phase 1b portion used a 3 + 3 design, and the phase 2 portion used a Simon two‐stage design. Eligible patients ages ≥60 years who had newly diagnosed AML with mutations in *TP53* and/or complex karyotype (≥3 cytogenetic abnormalities) received either 37 mg (group 1) or 25 mg (group 2) TP-0903 orally on days 1 to 21 with decitabine 20 mg/m^2^ on days 1 to 10 for up to three 28-day induction cycles, followed by up to 30 maintenance cycles in which decitabine dosing was reduced to days 1 to 5. The primary endpoint was complete remission (CR) by the end of six cycles of treatment.

**Results:**

The overall composite remission rate (CR/CR with incomplete count recovery/CR with hematologic improvement) was 33.3% in group 1 and 50.0% in group 2, with CR rates of 13.3% and 25%, respectively. The median overall survival for groups 1 and 2 was 7.6 and 7.5 months, respectively.

**Conclusions:**

The combination of TP-0903 and decitabine was reasonably tolerated and had activity in this patient population. Further research and novel treatment strategies are necessary to improve outcomes for patients with these high-risk subtypes of AML.

**Significance:**

Treatment options for AML with mutant *TP53* and/or complex karyotype are limited. In a phase 1b/2 clinical trial, TP-0903, a multikinase inhibitor, was well-tolerated and had activity comparable with other emerging therapies. Our results suggest that TP-0903 may offer insight and serve as a benchmark for the development of future agents leveraging similar strategies or scaffolds to improve outcomes in these intractable subtypes of AML.

## Introduction

The prognosis for older patients (≥60 years old) diagnosed with acute myeloid leukemia (AML) remains dismal, with a median overall survival (OS) less than 15 months (1–3), which is partially attributable to a higher incidence of poor-risk features (4). Among poor-risk genomic features, mutations in *TP53* and complex karyotype (≥3 cytogenetic abnormalities) each occur in around 20% of older patients with AML (5) versus rates of 10% or lower in younger patients (6, 7). These features commonly co-occur, with up to 70% of patients with complex karyotype also carrying a *TP53* mutation (8). Patients with mutant *TP53* or complex karyotype respond particularly poorly to standard induction chemotherapy (9), with a 30-day early death rate as high as 41% (10). Treatment with single-agent decitabine, a hypomethylating agent (HMA), has a reported complete remission (CR) rate of 21% (11) with a median OS of <1 year (11, 12). The addition of venetoclax to HMAs has resulted in rates of CR + CR with incomplete count recovery (CRi) of 55% but did not show any difference in OS when compared with HMA alone, with a median OS of approximately 5.2 months (12). New treatment strategies are urgently needed to improve outcomes for these patients.

*TP53* encodes p53, a transcription factor that acts as a tumor suppressor by helping to regulate the DNA damage response. The DNA damage response facilitates DNA repair during checkpoints in the cell cycle, including the G1 and G2 checkpoints (13). Cells with mutant or deleted *TP53* frequently have a defective G1 checkpoint and depend more on the G2 checkpoint (14). Inhibition of kinases involved in the G2 checkpoint, including aurora kinase A (AURKA) (; ref. 15), aurora kinase B (AURKB) (; ref. 16), and checkpoint kinase 1 (Chk1; ref. 17), induces cell death in *TP53*-mutated cancer cells, and Chk1 inhibition has induced synthetic lethality when combined with cisplatin (18) or inhibition of the aurora kinases (19) in cancers characterized by frequent *TP53* mutations. Kinase inhibitors have recently emerged as a potential therapy for *TP53*-mutated AML, including inhibitors of casein kinase II (20), Polo-like kinase 4 (21), and spleen tyrosine kinase (22). Based on these collective data, we hypothesized that inhibition of multiple kinases regulating the cell cycle could be an effective strategy to treat *TP53*-mutated AML.

TP-0903 is a small-molecule multikinase inhibitor that, despite originally being designed to inhibit AXL, inhibits AURKA/B, Chk1/2, and other cell-cycle regulators (23). TP-0903 monotherapy was well tolerated in clinical trials in solid tumors and chronic lymphocytic leukemia (ClinicalTrials.gov identifiers: NCT02729298 and NCT03572634). Based on compelling *in vitro* and *in vivo* activity in models of drug-resistant AML with both wild-type *TP53* (23) and mutant *TP53* (24), including additive activity with decitabine in two animal models of *TP53*-mutated AML, we sought to evaluate TP-0903’s clinical activity by conducting a phase 1b/2 study evaluating TP-0903 in combination with a 10-day decitabine regimen in patients ages 60 years and older who had newly diagnosed AML with mutant *TP53*, complex karyotype, or both.

## Patients and Methods

### Patients

This trial is a substudy of Beat AML Master Trial (ClinicalTrials.gov identifier: NCT03013998) conducted by Beat AML, a division of the Leukemia & Lymphoma Society, which has previously been described (5). The protocol was approved by both a central Institutional Review Board and the local Institutional Review Board at each participating center. The study was conducted in accordance with the Declaration of Helsinki and Good Clinical Practice Guidelines. All patients provided written informed consent before screening.

Consistent with Beat AML Master Trial (5), patients ages 60 years or older with newly diagnosed AML were enrolled and screened. Patients enrolled in this substudy had untreated AML with either a *TP53* mutation, complex karyotype, or both. Complex karyotype was defined as having three or more unrelated cytogenetic abnormalities. The dominant clone was determined using the Master Trial algorithm, and the variant allele frequency (VAF) cutoff for mutant *TP53* was 20% (5). Key inclusion criteria included an Eastern Cooperative Oncology Group performance status of 0 to 2, aspartate aminotransferase and alanine aminotransferase less than five times the upper limit of normal (ULN), total bilirubin less than two times the ULN, except for patients with known Gilbert syndrome, and creatinine clearance greater than 40 mL/minutes or serum creatinine less than 1.5 times the ULN. Key exclusion criteria included patients who were willing and able to receive induction chemotherapy (e.g., 7 + 3), patients with acute promyelocytic leukemia, active central nervous system involvement by AML, uncontrolled infection or known active human immunodeficiency virus, active hepatitis B or active hepatitis C infection, disseminated intravascular coagulopathy with active bleeding or signs of thrombosis, or patients who had previously received TP-0903 for myeloid malignancies. Patients who had received any investigational agent were required to wait five half-lives of the agent (or 4 weeks if the half-life was unknown) and until treatment-related toxicity had resolved to grade 1 or less prior to starting TP-0903. Patients were not allowed to have had prior HMA therapy for myelodysplastic syndrome in the setting of secondary AML. Complete inclusion and exclusion criteria are listed in Supplementary Table S1.

### Study design

**Figure 1:**
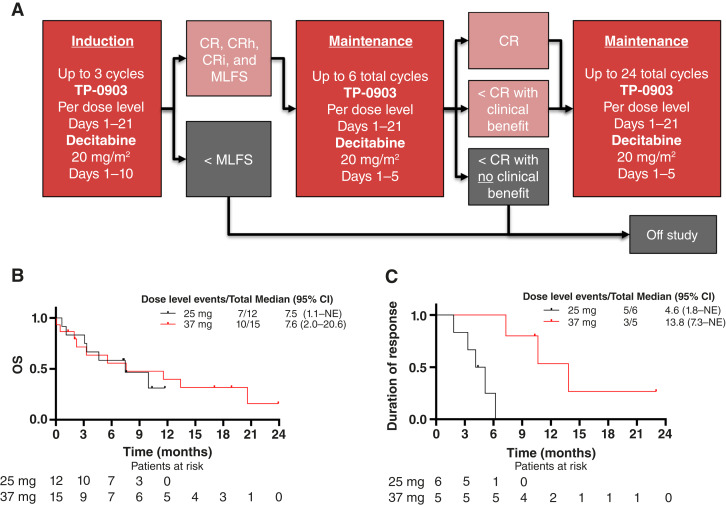
OS and duration of response. Patients were treated according to the (**A**) study schema. **B,** OS and (**C**) duration of response among patients who achieved CR + CRh + CRi. The median OS for all patients (*n* = 27) was 7.6 months (95% CI, 3.3–13.4) with a median follow-up of 9 months. Additional parameters are listed in Table 3.

This was an open-label, phase 1b/2, multicenter clinical study of TP-0903 in combination with decitabine. During an initial induction phase, patients were treated with TP-0903 orally once daily for days 1 to 21 and 20 mg/m^2^ decitabine intravenously for days 1 to 10 of up to three 28-day cycles. Patients who achieved CR, CR with hematologic improvement (CRh), CRi, or morphologic leukemia-free state (MLFS) were then treated with TP-0903 for days 1 to 21 and 20 mg/m^2^ decitabine for days 1 to 5 of up to six total cycles. After six total cycles of treatment, any patients who were experiencing clinical benefit (i.e., CR, CRh, CRi, or MLFS with benefit as defined by the treating physician) continued treatment with TP-0903 on days 1 to 21 and 20 mg/m^2^ decitabine for days 1 to 5 of up to 24 total cycles (Fig. 1A). The phase 1b portion of the study used a standard 3 + 3 dose-escalation design with a starting dose level 1 (DL1) of 37 mg oral daily TP-0903 (Supplementary Table S2) based on experience with 50 mg oral daily as a single agent in patients with solid tumors (25). Dose-limiting toxicities (DLT) were evaluated to establish the dose brought forward for an expansion cohort (phase 2). Seven patients were enrolled in the phase 1b study, which was completed in January 2021 with the decision to move forward with an expansion cohort of 37 mg oral daily. Across the phase 1b and phase 2 studies, 15 patients were enrolled and started on 37 mg TP-0903 oral daily on days 1 to 21 (group 1). However, in April 2021, enrollment was paused because of delayed count recovery noted in patients. One additional patient was enrolled at this time but was inadvertently started on 25 mg TP-0903 oral daily on days 1 to 21. A review of the phase 1b data revealed that, whereas the criteria for a DLT were not met, full recovery of counts was slow for five patients who completed three or more total cycles of therapy, including three patients who required >40 days for count recovery in the first cycle. Based on a review of the cumulative preclinical and clinical data, the review team concluded that observed delayed count recoveries may be attributable to the 37 mg dose of TP-0903 and that a lower dose may be more tolerable. In May 2021, an amendment was approved to allow enrollment of a new group 2 at 25mg oral daily on days 1 to 21 of TP-0903 (DL-1) in stage 2 of the phase 2 study. The flow of patient consent and enrollment in the phase 1b and phase 2 studies is illustrated in Supplementary Fig. S1.

### Outcomes

Clinical response was assessed according to the modified 2017 European LeukemiaNet AML criteria (26). The primary endpoint was achievement of CR within six cycles of therapy. Secondary endpoints included toxicity, duration of response, OS, and the proportion of eligible patients who effectively transitioned to allogeneic stem cell transplantation. Exploratory endpoints included pharmacokinetics (PK), correlative genetic, and biochemical data; correlations between morphologic remission, spectral flow cytometry, and mutational remission; and duration of remission or survival. The population evaluable for both safety and response included all patients who received any dose of either TP-0903 or decitabine. Adverse events (AE) were graded according to the NCI Common Terminology Criteria for Adverse Events version 4.0 (27).

### Correlative studies

#### Mutation analysis

Mutations in germline and serial bone marrow or blood samples were determined by targeted next-generation sequencing (NGS). NGS aligned read files are available in the NCBI’s Sequence Read Archive under accession PRJNA1236309. See Supplementary Methods for details.

#### PK

On days 1 and 10 of cycle 1, plasma samples were collected for PK analysis from a total of 25 patients. In the phase 1b study portion of the study, plasma was collected before administration of TP-0903 on day 10 and at 0.5, 1, 2, 4, 8, and 24 hours after administration of TP-0903 on days 1 and 10. In the phase 2 portion of the study, plasma was collected at 2, 4, and 24 hours after drug administration. A validated ultrahigh-performance LC-MS/MS method was developed and used to quantify TP-0903 and its four major metabolites (M2, M3, M4, and M6) in plasma.

TP-0903 and its metabolites M2, M3, M4, and M6 were quantified in plasma using solid phase extraction and LC-MS/MS in the Ohio State University Comprehensive Cancer Center Pharmacoanalytical Shared Resource. See Supplementary Methods for details.

#### Spectral flow

Spectral flow cytometry was completed on peripheral blood samples obtained at pretreatment and on day 10 of treatment from patients enrolled to the phase 1b portion of the study, as previously described (28). In brief, Ficoll-enriched peripheral blood mononuclear cells were stained for external markers, fixed, permeabilized, and then stained for p-H2AX (Thermo Fisher Scientific; AB_2896984). Sample analysis was performed in the Ohio State University Comprehensive Cancer Center Flow Cytometry Shared Resource and Immune Monitoring & Discovery Platform using the Cytek Aurora. Data were analyzed using FCS Express version 7 (*De Novo* Software).

### Statistical methods

The phase 1b portion of the study used a standard 3 + 3 design with dose-escalation based on DLT. The phase 2 portion utilized a Simon optimal two-stage design testing the null hypothesis that the true CR rate is 21%, based on published experience with decitabine (11), against a one-sided alternative hypothesis of a CR rate of 45%. In the first stage, if four or more of 12 patients achieved CR, then an additional 21 patients would be enrolled for a total of 33 patients. At the end of the study, if 12 or more of all 33 patients achieved CR, then the null hypothesis would be rejected. This design yielded a one-sided type 1 error rate of 2.5% and power of 80% when the true response rate is 45%.

After the phase 2, stage 1 portion of the study was restarted at 25 mg/day of TP-0903 on days 1 to 21 (See “Study design”), a similar Simon optimal two-stage design was used to inform a goal of enrolling 33 patients to receive 25 mg/day TP-0903 in group 2 (i.e., in addition to patients who had already been enrolled to group 1 with planned analyses independent of group 1). Of the 12 patients treated with 25 mg/day TP-0903 (i.e., the 11 enrolled to 25 mg/day and one patient enrolled to 37 mg/day that received 25 mg/day), only three achieved CR. Thus, the requisite CR was not met to proceed to stage 2, and the phase 2 study was terminated.

Demographics were summarized by descriptive statistics. Duration of response was calculated from the date when composite CR was achieved to death due to any cause, censoring patients who remained alive and relapse-free at the time of the last follow-up. OS was calculated from treatment start to all-cause death, censoring patients who were alive at the time of the last follow-up. Kaplan–Meier methodology was used to assess OS and duration of response.

### Data availability

NGS aligned read files are available in the NCBI’s Sequence Read Archive under accession PRJNA1236309. The data generated in this study are available upon reasonable request from the corresponding author.

## Results

### Baseline patient characteristics

Thirty patients were enrolled in this trial between August 2020 and February 2022, of whom 27 received treatment with decitabine and TP-0903 (Supplementary Fig. S1). Fifteen patients were treated with 37 mg TP-0903/day on days 1 to 21 (group 1), and 12 patients were treated with 25 mg TP-0903/day on days 1 to 21 (see “Patients and Methods”). Baseline characteristics and demographics for all patients and each group are displayed in Table 1. The median age at diagnosis in group 1 was 73 (range 62–84) and in group 2 was 70 (range 62–81) years. Most patients in both group 1 and group 2 were male (60% and 67%, respectively) and Caucasian (93% and 92%, respectively), which is generally representative of trends observed in the diagnosis of AML (Supplementary Table S3). Only one patient enrolled in the study had treatment-related AML and was treated in group 1 (7%).

**Table 1:** Baseline characteristics and demographics

Characteristic	Group 1 37 mg (*N* = 15)	Group 2 25 mg (*N* = 12)	Overall (*N* = 27)
Age, years	​	​	​
Median (range)	73 (62–84)	70 (62–81)	72 (62–84)
Gender, no. (%)	​	​	​
Female	6 (40)	4 (33.3)	10 (37.0)
Male	9 (60)	8 (66.7)	17 (63.0)
Race, no. (%)	​	​	​
Caucasian	14 (93.3)	11 (91.7)	25 (92.6)
African American	0 (0)	1 (8.3)	1 (3.7)
Asian	1 (6.7)	0 (0)	1 (3.7)
Performance status, no. (%)	​	​	​
0	3 (20)	7 (58.3)	10 (37.0)
1	9 (60)	1 (8.3)	10 (37.0)
2	3 (20)	4 (33.3)	7 (25.9)
Hemoglobin, g/L	​	​	​
Median (range)	82 (69–124)	80.5 (66–90)	82 (66–124)
Platelets, 10^3^/μL	​	​	​
Median (range)	36 (8–464)	44 (20–190)	42.5 (8–464)
Unknown	0	1	1
WBC, 10^3^/μL	​	​	​
Median (range)	3.6 (0.7–37.8)	5.4 (0.6–26.8)	4.5 (0.6–37.8)
Unknown	​	1	1
WBC ≥50, no. (%)	0 (0)	0 (0)	0 (0)
ANC, 10^3^/μL	​	​	​
Median (range)	0.4 (0–13.4)	0.3 (0.1–6.5)	0.4 (0–13.4)
Blood blasts, %	​	​	​
Median (range)	14 (0–96)	28.1 (0.9–87)	26.5 (0–96)
Not assessed/unknown	2	1	3
Bone marrow blasts, %	​	​	​
Median (range)	34 (18–91)	57 (11–86)	47 (11–91)
Treatment-related AML, no. (%)	​	​	​
No	14 (93.3)	12 (100)	26 (96.3)
Yes	1 (6.7)	0 (0)	1 (3.7)
Complex cytogenetics, no. (%)	​	​	​
Absent	0 (0)	0 (0)	0 (0)
Present	14 (100)	12 (100)	26 (100)
Not assessed/unknown	1	0	1
TP53, no. (%)	​	​	​
WT (VAF <20%)	4 (26.7)	3 (25.0)	7 (25.9)
Mutated (VAF ≥20%)	11 (73.3)	9 (75.0)	20 (74.1)
FLT3-ITD, no. (%)	​	​	​
Absent	15 (100)	12 (100)	27 (100)
Present	0 (0)	0 (0)	0 (0)
FLT3-TKD, no. (%)	​	​	​
WT (VAF <20%)	15 (100)	12 (100)	27 (100)
Mutated (VAF ≥20%)	0 (0)	0 (0)	0 (0)
FLT3 (other), no. (%)	​	​	​
WT (VAF <20%)	14 (93.3)	12 (100)	26 (96.3)
Mutated (VAF ≥20%)	1 (6.7)	0 (0)	1 (3.7)
TET2, no. (%)	​	​	​
WT (VAF <20%)	13 (86.7)	10 (83.3)	23 (85.2)
Mutated (VAF ≥20%)	2 (13.3)	2 (16.7)	4 (14.8)

Abbreviations: ANC, absolute neutrophil count; WBC, white blood cell; WT, wild-type.

All treated patients had complex karyotypes. The majority of patients had mutant *TP53* (VAF >20%), including 11 patients in group 1 (73%) and nine patients in group 2 (75%). Two patients in group 1 (13%) and two patients in group 2 (17%) had mutations in Tet methylcytosine dioxygenase 2 (*TET2*) (VAF >20%). Other mutations evaluated in the Beat AML screening algorithm (5) were rare. No enrolled patients had *FLT3*-ITD or mutations in *NPM1*, *IDH1*, *IDH2*, or *WT1*.

### Safety

Seven patients were enrolled and treated at DL1 in the phase 1b portion of the study. Although the first three patients had no DLTs, they experienced delayed count recovery; therefore, it was decided to enroll an additional three patients; of note, a total of seven patients were enrolled to the phase 1b study, as one patient was not evaluable for DLT due to noncompliance with the DLT observation period. No DLTs were observed in the six evaluable patients at DL1. Based on these data, 37 mg TP-0903 was decided to be the dose brought forward for an expansion cohort, and eight additional patients were quickly enrolled and started on 37 mg TP-0903 oral daily on days 1 to 21 (group 1). After the decision to expand (to stage 1 of the phase 2 study) at this dose level, rather than to dose-escalate, further analyses of myelosuppression, based on the data from five evaluable patients in the phase 1b portion that completed three cycles of therapy and Beat AML’s experience, informed the decision to reduce the dose to 25 mg and restart enrollment to stage 1 of the phase 2 study. Twelve patients were treated in group 2, and the study did not advance to stage 2.

For both groups 1 and 2, Table 2 displays a summary of observed grade 3+ treatment-related AEs, and Supplementary Table S4 lists all observed AEs (all-cause, all grade). Table 2 reports the number of patients that experienced a grade 3+ treatment-related AE during any cycle, with the higher-grade AE considered if the AE occurred multiple times. Seven patients in each group had treatment-related grade 3+ AEs (47% in group 1; 58% in group 2). Most grade 3+ treatment-related AEs were hematologic. The most common grade 3+ treatment-related AEs in group 1 were neutropenia (33%) and thrombocytopenia (27%), whereas the most common grade 3+ treatment-related AEs in group 2 were neutropenia (50%), lymphocyte count decrease (42%), febrile neutropenia (33%), leukopenia (33%), and thrombocytopenia (25%). Nonhematologic grade 3+ treatment-related AEs were rare, and, in group 1, included stomatitis (6%) and fatigue (6%) and, in group 2, pneumonia (8%) and decreased appetite (8%). Other common grade 1 to 2 treatment-related AEs (any grade) were gastrointestinal and in group 1 included nausea (47%) and diarrhea (33%) and in group 2 included nausea (42%), decreased appetite (25%), and diarrhea (25%). Notably, only one patient had a serious AE related to decitabine (grade 3 pneumonia), and no patients had serious AEs related to TP-0903.

**Table 2:** Grade ≥3 treatment-related AEs

Treatment-related AEs Grade ≥3, no. (%)	Group 1 37 mg/day (*n* = 15)	Group 2 25 mg/day (*n* = 12)	Overall (*n* = 27)
Blood and lymphatic system disorders		​	​
Neutropenia	5 (33.3)	6 (50)	11 (40.7)
Thrombocytopenia	4 (26.7)	3 (25)	7 (25.9)
Leukopenia	3 (20)	4 (33.3)	7 (25.9)
Anemia	2 (13.3)	1 (8.3)	3 (11.1)
Febrile neutropenia	0 (0)	4 (33.3)	4 (14.8)
Lymphocyte count decreased	0 (0)	5 (41.7)	5 (18.5)
Gastrointestinal disorders	​	​	​
Stomatitis	1 (6.3)	0 (0)	1 (3.7)
General disorders	​	​	​
Fatigue	1 (6.3)	0 (0)	1 (3.7)
Infections	​	​	​
Pneumonia	0 (0)	1 (8.3)	1 (3.7)
Metabolism and nutrition disorders		​	​
Decreased appetite	0 (0)	1 (8.3)	1 (3.7)

### Response to treatment

The median duration of treatment in group 1 was 1.5 months (range 0.1–16.4 months) with two cycles of TP-0903 (range 1–16) and in group 2 was 3.2 months (range 0.2–7.0 months) with three cycles of TP-0903 (range 1–6; Supplementary Table S5). In group 1, the most common reasons for treatment discontinuation were withdrawal of consent (27%), AEs (13%), treatment failure (13%), recurrence after a response (13%), and stem cell transplantation (13%). In group 2, the most common reasons for treatment discontinuation were treatment failure (25%), AEs (17%), recurrence after a response (17%), and disease progression (17%).

Treatment response, response duration, and OS are listed in Table 3. Of the 15 patients in group 1, two achieved the primary endpoint of CR (13%), and three patients achieved CRh (20%) with up to six cycles of treatment. Otherwise, one patient had MLFS (7%), five had stable disease (SD; 33%), two had treatment failure (13%), and two stopped treatment within 2 days because of death or consent withdrawal (13%). Therefore, the overall composite remission rate (CR/CRi/CRh) was 5/15 = 33.3% [95% confidence interval (CI), 11.8%–61.6%]. Of the 12 patients in group 2, three achieved CR (25%), one achieved CRh (8%), and two achieved CRi (17%) within six cycles. Additionally, one patient had MLFS (8%), three had SD (25%), and two stopped treatment within four and 5 days, respectively, because of AEs (17%). Therefore, the overall composite remission rate (CR/CRi/CRh) was 6/12 = 50% (95% CI, 21.1%–78.9%). If we consider only patients with a *TP53* mutation (*n* = 20), irrespective of enrollment to group 1 or 2, the overall composite remission rate (CR/CRi/CRh) was 9/20 = 45% (95% CI, 23.1%–68.5%) for these patients.

**Table 3:** Treatment response, response duration, and OS table

Treatment outcomes	Group 1 37 mg/day (*n* = 15)	Group 2 25 mg/day (*n* = 12)	Overall (*n* = 27)	*TP53*-mutated patients (*n* = 20)
Best response by 6 cycles, no. (%)	​	​	​	​
CR	2 (13.3)	3 (25)	5 (18.5)	5 (25)
CRh	3 (20)	1 (8.3)	4 (14.8)	4 (20)
CRi	0 (0)	2 (16.7)	2 (7.4)	0 (0)
MLFS	1 (6.7)	1 (8.3)	2 (7.4)	2 (10)
SD	5 (33.3)	3 (25)	8 (29.6)	4 (20)
Treatment failure	2 (13.3)	0 (0)	2 (7.4)	1 (5)
NE	2 (13.3)	2 (16.7)	4 (14.8)	4 (20)
Overall composite remission rate, (CR/CRh/CRi), no. (%, 95% CI)	5 (33.3, 11.8–61.6)	6 (50, 21.1–78)	11 (40.7, 22.4–61.2)	9 (45, 23.1–68.5)
Overall response rate, (CR/CRh/CRi/MLFS), no. (%, 95% CI)	6 (40, 16.3–67.7)	7 (58.3, 27.7–84.8)	13 (48.2, 28.7–68.1)	11 (55, 31.5–76.9)
OS	​	​	​	​
No. of events (deaths)	10	7	17	13
Med., months (95% CI)	7.6 (2.0–20.6)	7.5 (1.1 – NE)	7.6 (3.3–13.4)	10.0 (2.0–20.6)
Med. FU, months (range)	17.1 (1.3–23.9)	7.6 (7.2–11.7)	9 (1.3–23.9)	7.6 (1.3–23.9)
Response duration	​	​	​	​
No. of events	3	5	8	6
Med., months (95% CI)	13.8 (7.3–NE)	4.6 (1.8–NE)	7.3 (3.3–13.8)	10.6 (3.3–NE)
Med. FU, months (range)	16.7 (10.3–23)	4.4 (4.4–4.4)	10.3 (4.4–23)	10.3 (4.4–23)

Abbreviations: ANC, absolute neutrophil count; FU, follow-up; NE, not evaluable.

Among patients who achieved CR/CRi/CRh, the median duration of response was 13.8 months [95% CI, 7.3–not reached (NR)] in group 1 and 4.6 months (95% CI, 1.8–NR) in group 2 (Fig. 1B; Table 3). With a median duration of follow-up of 17.1 months in group 1 and 7.6 months in group 2, the median OS was 7.6 months (95% CI, 2.0–20.6) in group 1 and 7.5 months (95% CI, 1.1–NR) in group 2 (Fig. 1C; Table 3). Remarkably, with a median duration of follow-up of 7.6 months, the median OS for patients that had *TP53* mutations was 10 months (95% CI, 2.0–20.6; Table 3).

### Serial sequencing

**Figure 2:**
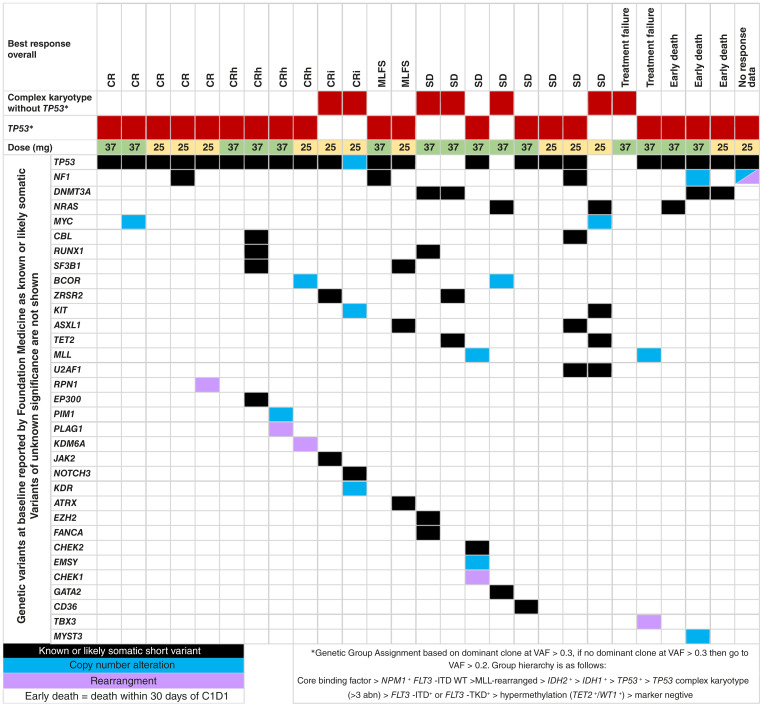
Sequencing data. Baseline mutations vs. response.

**Figure 3:**
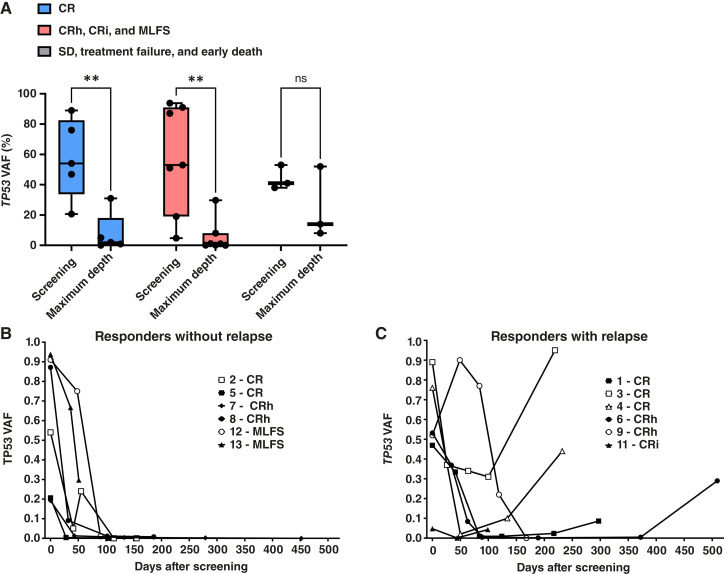
*TP53* VAF. **A,***TP53* VAF by best response. *TP53* VAF over time in patients that achieved CR/CRh/CRi/MLFS (**B**) without relapse on study or (**C**) with relapse while on study. Patients who received 37 mg TP-0903 are represented by closed symbols, whereas patients who received 25 mg TP-0903 are represented by open symbols. **, *P* < 0.01; ns, not significant.

Patients were enrolled to this substudy of Beat AML Master Trial based on NGS performed by Foundation Medicine, Inc.; baseline mutations at enrollment versus response are found in Fig. 2. Five of the 14 patients who had SD, treatment failure, or early death had no *TP53* mutation, whereas all 13 patients who achieved CR/CRh/CRi/MLFS had a *TP53* mutation or copy-number alteration. To track mutation clearance or the emergence of new mutations, we performed serial sequencing with samples obtained from 20 patients (Supplementary Fig. S2). In general, the emergence of new mutations was rare. Among patients who had a *TP53* mutation at screening and at least one additional sample, the *TP53* VAF was significantly decreased in patients who achieved CR (*n* = 5; *P* < 0.01) and CRh/CRi/MLFS (*n* = 7; *P* < 0.01) but was not significantly changed in patients who had SD, treatment failure, or early death (*n* = 3; Fig. 3A). In patients who achieved CR/CRh/CRi/MLFS, the *TP53* VAF decreased over time (Fig. 3B). In patients who relapsed after achieving CR/CRh/CRi, the *TP53* VAF increased (Fig. 3C).

### PK

PK parameters for TP-0903, its four active metabolites (M2, M3, M4, and M6), and the sum of species are summarized in Supplementary Table S6. In group 1, which received 37 mg TP-0903, the median TP-0903 C_max_ and AUC_0–24h_ were 3.3 ng/mL (range 0.71–23) and 36 hours × ng/mL (range 12–340) on day 1 and 13 ng/mL (range 1.8–45) and 210 hours × ng/mL (range 37–690) on day 10 (i.e., steady state). In group 2, which received 25 mg TP-0903, the median TP-0903 C_max_ and AUC_0–24h_ were 2.4 ng/mL (range 0.22–12) and 42 hours × ng/mL (range 4–181) on day 1 and 14 ng/mL (range 1.2–44) and 260 hours × ng/mL (110–690) on day 10. TP-0903 exposure was higher at steady state versus after a single dose; based on the median AUC, TP-0903 had a drug accumulation ratio of six in both groups. There was no obvious difference in PK between groups 1 and 2, likely due in part to highly variable TP-0903 PK with >14-fold variation in steady-state TP-0903 plasma exposure (i.e., collected on cycle 1, day 10 before TP-0903 was received) in each group. PK parameters were not associated with response or toxicity. There was no significant difference in steady-state TP-0903 plasma exposure (C_ss_ Min on Day 10) between patients who responded and those who did not respond (Supplementary Fig. S3A). Patients with steady-state TP-0903 plasma exposure above the median did not have a greater decrease in absolute neutrophil count (Supplementary Fig. S3B) or platelets (Supplementary Fig. S3C).

### Correlative biology studies

Based on the observation that TP-0903 upregulates p-H2AX, a marker of DNA damage, in *TP53*-mutated cell lines (24), we hypothesized that p-H2AX could serve as a clinical biomarker for TP-0903 activity. Analysis of peripheral blood from days 1 and 10 of cycle 1 was performed by spectral flow cytometry. The mean fluorescence intensity was measured for p-H2AX, and then values at day 10 were normalized using baseline values collected on day 1 (Supplementary Fig. S4). There was no significant change in levels of p-H2AX in patients who achieved CR (*n* = 2), CRh/CRi/MLFS (*n* = 8), or those with SD, treatment failure, or early death (*n* = 7).

## Discussion

Patients with *TP53*-mutated AML have a poor prognosis that is even more exceptionally dismal in the presence of complex karyotype. For *TP53*-mutated patients, the median OS ranges from 4 to 9 months with intensive chemotherapy (29), 7.9 months with azacitidine (30), or up to 12.7 months with decitabine (31). Based on these outcomes, there has been intense interest in developing investigational therapies for *TP53*-mutated AML (32). In this context, venetoclax in combination with azacitidine has been a promising frontline option in older or medically unfit patients with AML, with the median OS as high as 17.5 months; however, the median OS in *TP53*-mutated patients with poor-risk cytogenetics treated with venetoclax and azacitidine was similar to azacitidine alone at only 5.2 months (33). The median OS of patients with mutant *TP53* treated with entospletinib, an inhibitor of SYK, combined with decitabine was only 6.5 months (22). Results with immunotherapies have also been poor, with a median OS of 9.8 months for *TP53*-mutated patients treated with magrolimab and azacitidine (34). Whereas eprenetapopt represented one of the most promising therapies to target mutant p53 with CR rates as high as 17%, the median OS with eprenetapopt combined with azacitidine was still less than 1 year (35, 36), which was not improved by the addition of venetoclax (37). New treatment strategies are needed to improve outcomes for patients with these intractable AML subtypes.

In this study, we have reported the results of a clinical trial assessing the addition of TP-0903, an investigational multikinase inhibitor, to decitabine treatment for patients with AML with mutant *TP53*, complex karyotype, or both. In general, the combination was tolerated well, with a limited number of grade 3+ treatment-related AEs that were mostly hematologic and consistent with the known toxicity profile of TP-0903 (25) and decitabine. In fact, there were no serious AEs attributable to TP-0903 in our trial. Observed response rates and OS were consistent with other emerging therapies in these high-risk subtypes of AML.

This study tested the null hypothesis that the true CR rate with the addition of TP-0903 is 21% against an ambitious one-sided alternative hypothesis of a CR rate of 45%. The 21% CR rate was based on a subgroup analysis of patients with AML with mutant *TP53* who received a 10-day course of decitabine; it is unclear how many patients in this subgroup had complex karyotype AML (11). However, all of the patients enrolled in the present study had complex karyotype AML with or without *TP53* mutations. Given the well-established notion that patients with both complex karyotype and mutant *TP53* have a particularly dismal prognosis (6, 9), it is tempting to speculate that the CR rate expected in the population enrolled in the present trial, when treated with a 10-day course of decitabine alone, is lower than our original prediction, which was based on patients with AML with mutant *TP53* who may or may not have had complex karyotype. Indeed, a recently published substudy of Beat AML Master Trial with inclusion/exclusion criteria similar to the present study recently reported a 17.1% CR + CRi rate in patients with complex karyotype AML with a *TP53* mutation treated with entospletinib and a 10-day course of decitabine (22); the overall composite remission rates (CR + CRh + CRi) of 33.3% and 50% observed in groups 1 and 2 of the present trial, respectively, compare favorably with our historic results with entospletinib plus decitabine and imply that the addition of TP-0903 to decitabine improved upon entospletinib plus decitabine treatment (and, ostensibly, upon expected outcomes with decitabine alone). Nonetheless, the observed overall composite remission rate is similar to the 55% CR + CRi rate observed in patients with mutant *TP53* who were treated with venetoclax plus azacitidine (12), and the median OS in the present trial (7.6 months) was similar to that of other leading investigational therapies for *TP53*-mutated AML. For patients with mutant *TP53* who received 25 mg, the true CR rate was 33.3% (3 of 9), which is similar to outcomes achieved with venetoclax plus HMA (38). The achievement of outcomes similar to leading therapies is notable considering that clinical experience with TP-0903 is limited and the patient population enrolled in the present trial was relatively broad and included patients without *TP53* mutations who counterintuitively responded more poorly to TP-0903 than patients with *TP53* mutations.

This trial sought to test whether TP-0903’s inhibition of multiple cell cycle kinases could effectively treat AML with mutant *TP53* and/or complex karyotype. We initially tested the concept that inhibition of multiple kinases involved in the cell cycle could be an effective treatment strategy in drug-resistant and *TP53*-mutated AML by testing TP-0903 in *in vitro* and *in vivo* models (23, 24). We first studied TP-0903 in preclinical models of AML with drug-resistant mutations other than *TP53*, including *FLT3*, *IDH2*, and *NRAS* (23). However, consistent with the design of Beat AML Master Trial and the general standard of care, patients with other targetable mutations (e.g., *FLT3*-ITD) with complex karyotype and/or mutant *TP53* were effectively excluded from the present trial. Follow-up studies found TP-0903 to be highly effective in preclinical models of *TP53*-mutated AML; TP-0903 was found to be more effective than decitabine and to have additive activity with decitabine *in vitro* and *in vivo*. Indeed, based on the available preclinical data (24), the finding that none of the five patients without a *TP53* mutation achieved CR, and the relatively favorable median OS for *TP53*-mutated patients in this trial (10 months), TP-0903 seems to be particularly effective in *TP53* mutant AML; this implies that the already favorable complete remission rate could have been even better had we only targeted patients with *TP53*-mutated AML. Consistent with this thesis, there was a correlation between response and clearance of *TP53* VAF among patients with *TP53* mutations. Indeed, serial sequencing demonstrated *TP53* VAF clearance over time with sustained VAF clearance in responders that increased only upon relapse. In this context, it is worth mentioning that *TP53* mutations are enriched in patients with therapy-related AML (39), suggesting that these mutations are particularly resistant to clearance, especially by induction chemotherapy. Thus, it is tempting to speculate that additional patients with *TP53*-mutated AML, including those with additional targetable comutations, therapy-related AML, and/or relapsed/refractory AML, might respond favorably to TP-0903, although this was not tested in this trial. However, whereas the ability of TP-0903 to inhibit multiple kinases could contribute to its activity in high-risk AML with multiple druggable mutations, it remains unclear which kinases, when inhibited, contribute to TP-0903’s activity, toxicity, or both.

TP-0903’s complicated PK and pharmacodynamic profile represents a challenge for the development of this agent. TP-0903 has highly variable PK with at least four active metabolites and ostensibly lacks dose proportionality. Plasma exposure of TP-0903 was highly variable, with substantial overlap in plasma exposure in groups 1 (37 mg) and 2 (25 mg) and >25-fold variation in the steady-state C_max_ of parent TP-0903 in both groups. Nonetheless, given that preclinical data suggest that TP-0903 and its metabolites may be enriched within the bone marrow (23), plasma levels may not be informative when determining concentrations at the site of action. Consistent with this idea, we did not observe a correlation between TP-0903 plasma exposure and response or toxicity (Supplementary Fig. S3). Indeed, regardless of the nearly 50% difference in dose, group 1 (37 mg) and group 2 (25 mg) had similar numbers of patients who experienced grade ≥3 treatment-related AEs (Table 2). Whereas a notable difference in the median duration of response was observed in the limited number of responders within group 1 (13.8 months; five patients) and group 2 (4.6 months; six patients), the median OS (Table 3) was similar in both groups and not substantially different from expected OS with standard of care.

Considering the lack of available effective therapies for patients with AML with *TP53* mutations and/or complex karyotype, the results of this clinical trial suggest that TP-0903 may offer insight and serve as a benchmark for the development of future agents leveraging similar strategies or scaffolds, which could improve outcomes for patients with *TP53* mutant and/or complex karyotype AML. Nonetheless, outcomes with TP-0903 were poor, consistent with the current standard of care, which supports the idea that additional efforts are direly needed to improve outcomes for this patient population.

## Supplementary Material

Supplementary Table S1 Inclusion and Exclusion Criteria

Supplementary Table S2 Dose Escalation Table

Supplementary Table S3 Representativeness of Study Participants

Supplementary Table S4 All-Cause Adverse Events

Supplementary Table S5 Treatment Completion and Reason Off Treatment

Supplementary Table S6 TP-0903 and metabolite pharmacokinetic parameters on days 1 and 10 of course 1

Supplementary Table S7 Research Resource Identifiers (RRID)

Supplementary Methods Supplementary Methods

Supplementary Figure S1 Consort diagram

Supplementary Figure S2 Sequencing data. Serial sequencing versus response.

Supplementary Figure S3 Pharmacokinetic exposure versus toxicity/response. (A) Steady-state plasma levels of TP-0903 parent drug in non-responders and responders (CR, CRh, CRi, MLFS). Percent decrease in (B) absolute neutrophil count (ANC) or (C) platelets in patients with a steady state plasma level above or below the median.

Supplementary Figure S4 p-H2AX as a pharmacodynamic biomarker for TP-0903. Spectral flow cytometry was performed to assess p-H2AX using peripheral blood collected at day 1 (i.e., baseline) and day 10, and change from screening was calculated for patients that achieved CR, patients that achieved CRh, CRi, or MLFS, and non-responders.
