# Supplementary material for: A Phase 1b/2 Study of TP-0903 and Decitabine Targeting Mutant TP53 and/or Complex Karyotype in Patients with Untreated Acute Myeloid Leukemia ≥Age 60 Years
Source: Cancer Res Commun. 2025 Jul 14;5(7):1129–39. doi: 10.1158/2767-9764.CRC-25-0091 (PMC12257073; doi:10.1158/2767-9764.CRC-25-0091)
Supplement: Supplementary Table S1 — Inclusion and Exclusion Criteria [file crc-25-0091_supplementary_table_s1_suppst1.docx]

**Supplementary Table S1. Inclusion and Exclusion Criteria**

| **Inclusion Criteria** | **Exclusion Criteria** |
| --- | --- |
| 1. Adults, age ≥ 60 years at the time of diagnosis with untreated AML that has either a TP53 mutation with or without complex karyotype (complex karyotype defined as ≥ 3 abnormalities) or complex karyotype without TP53 mutation. No chemotherapy for AML outside of hydroxyurea for treatment of leukostasis or All-trans Retinoic Acid (ATRA) for initially suspected Acute Promyelocytic Leukemia (APL) (that is ruled out) is allowed. Prior therapy for myelodysplastic syndrome is (MDS) allowed except for hypomethylating agents. 2. Patients must be able to understand and provide written informed consent. 3. Eastern Cooperative Oncology Group (ECOG) performance status 0, 1, or 2. 4. Aspartate aminotransferase (AST) < 2.5 x upper limit of normal (ULN), alanine aminotransferase (ALT) < 2.5 x ULN, and total bilirubin < 1.5 x ULN (except for patients with known Gilbert’s syndrome) for the local laboratory. If due to disease, higher values may be approved after discussion with medical monitor. 5. Adequate renal function as defined by calculated creatinine clearance (according to the Cockcroft-Gault equation) > 40 mL/min OR serum creatinine < 1.5 x the ULN for the local laboratory. 6. Female patients of childbearing potential must agree to use 2 forms of contraception from screening visit until 120 days following the last dose of study treatment. Should a woman become pregnant or suspect she is pregnant while she or her partner is participating in this study, she should inform her treating physician immediately. Male patients having intercourse with females of childbearing potential must agree to abstain from heterosexual intercourse or have their partner use 2 forms of contraception from screening visit until 120 days after the last dose of study treatment. They must also refrain from sperm donation from screening visit until 120 days following the last dose of study treatment. | 1. Patients willing and able to receive intensive induction chemotherapy, such as 7+3. 2. Isolated myeloid sarcoma (meaning, patients must have blood or marrow involvement with AML to enter the study). 3. Acute promyelocytic leukemia (FAB M3). 4. Active central nervous system (CNS) involvement by AML. 5. Clinical signs/symptoms of leukostasis. 6. Known active Human Immunodeficiency Virus (HIV), active hepatitis B or active hepatitis C infection. 7. Disseminated intravascular coagulopathy with active bleeding or signs of thrombosis. 8. Patients who have received an investigational agent (for any indication) within 5 half-lives of the agent and until toxicity from this has resolved to grade 1 or less; if the half-life of the agent is unknown, patients must wait 4 weeks prior to first dose of study treatment. An investigational agent is one for which there is no approved indication by the United States (US) FDA. 9. Prior treatment with TP-0903 for myeloid malignancies. 10. Patients with psychological, familial, social, or geographic factors that otherwise preclude them from giving informed consent, following the protocol, or potentially hamper compliance with study treatment and follow-up. 11. Patients who are otherwise felt unable to comply with the protocol, in the opinion of the investigator. 12. Any other significant medical condition, including psychiatric illness or laboratory abnormality that would preclude the patient participating in the trial or would confound the interpretation of the results of the trial. 13. Patients with the following will be excluded: uncontrolled intercurrent illness including, but not limited to, symptomatic (New York Heart Association (NYHA) Class III or IV) congestive heart failure, unstable angina pectoris, serious cardiac arrhythmia, myocardial infarction as presentation of AML, severe uncontrolled ventricular arrhythmias, or electrocardiographic evidence of acute ischemia or active conduction system abnormalities. Patients with medical comorbidities that will preclude safety evaluation of the combination should not be enrolled. 14. Patients with QTcF > 480 ms at screening; patients with right, left, or partial bundle branch blocks that may confound interpretation of this reading are excluded from this provided they lack history of primary arrhythmic events and are cleared by cardiology for enrollment in the trial. 15. Patients with uncontrolled infection shall not be enrolled until infection is treated and controlled. |
