# Supplementary material for: A Phase 1b/2 Study of TP-0903 and Decitabine Targeting Mutant TP53 and/or Complex Karyotype in Patients with Untreated Acute Myeloid Leukemia ≥Age 60 Years
Source: Cancer Res Commun. 2025 Jul 14;5(7):1129–39. doi: 10.1158/2767-9764.CRC-25-0091 (PMC12257073; doi:10.1158/2767-9764.CRC-25-0091)
Supplement: Supplementary Table S2 — Dose Escalation Table [file crc-25-0091_supplementary_table_s2_suppst2.docx]

**Supplementary Table S2. Dose Escalation Table**

| **Dose Level** | **TP-0903 (mg/day PO)** | **Decitabine (mg/m^2^ IV)**  **Induction 1, 2, 3: Days 1-10**  **Continued Therapy: Days 1-5** |
| --- | --- | --- |
| -2 | 12 | 20 |
| -1 | 25 | 20 |
| 1 | 37 | 20 |
| 2 | 50 | 20 |
