# Supplementary material for: A Phase 1b/2 Study of TP-0903 and Decitabine Targeting Mutant TP53 and/or Complex Karyotype in Patients with Untreated Acute Myeloid Leukemia ≥Age 60 Years
Source: Cancer Res Commun. 2025 Jul 14;5(7):1129–39. doi: 10.1158/2767-9764.CRC-25-0091 (PMC12257073; doi:10.1158/2767-9764.CRC-25-0091)
Supplement: Supplementary Table S3 — Representativeness of Study Participants [file crc-25-0091_supplementary_table_s3_suppst3.docx]

**Supplementary Table S3. Representativeness of Study Participants**

| **Cancer type(s)/subtype(s)/stage(s)/condition** | Acute Myeloid Leukemia (AML) |
| --- | --- |
| **Considerations related to:** | |
| **Sex** | AML is more commonly diagnosed in males, which was seen in this trial population. The SEER 5-year (2018-2022) age-adjusted incidence rate of AML was 3.6 per 100,000 in females and 5.1 per 100,000 in males. |
| **Age** | The median age at diagnosis is 69 years. This clinical study was designed for older AML patients 60 years and older. The median age for this study is 72 years, which is representative. |
| **Race/ethnicity** | AML is more commonly diagnosed in Caucasians, which was seen in this trial population. The SEER 5-year (2018-2022) age-adjusted incidence rate per 100,000 were 4.5 in Non-Hispanic White, 3.8 in Non-Hispanic Black, 3.5 in Non-Hispanic Asian/Pacific Islanders, 3.7 in Non-Hispanic American Indian / Alaskan Native, and 3.6 in Hispanic (any race). |
| **Geography** | In the United States, there will be an estimated 22,010 AML cases diagnosed in 2025. The age-adjusted incidence rates per 100,000 in 2021 at our clinical trial research sites were, respectively, Ohio (4.2), Oregon (4.0), Maryland (NA), Texas (3.9), Utah (4.6), Kansas (NA), North Carolina (4.3), Pennsylvania (4.4), and Florida (NA). However, our research sites generally only capture large metropolitan areas. |
| **Other considerations** | AML patients for this trial were also required to have mutant *TP53* and/or complex karyotype, which occurs in approximately ~10% of AML and limited our ability to achieve diversity. |
| **Overall representativeness of this study** | Minorities are generally underrepresented in AML clinical trials compared to the incidence rate of AML, and this was the case here. However, the small size of this early-stage clinical trial, with only 27 patients enrolled overall, made it challenging to achieve a complete representation of diversity. |

^SEER*Explorer: An interactive website for SEER cancer statistics [Internet]. Surveillance Research Program, National Cancer Institute; 2025 Apr 16. [cited 2025 May 15]. Available from: <https://seer.cancer.gov/statistics-network/explorer/>. Data source(s): SEER Incidence Data, November 2024 Submission (1975-2022), [SEER 21 registries](https://seer.cancer.gov/registries/terms.html).
