# Supplementary material for: A Phase 1b/2 Study of TP-0903 and Decitabine Targeting Mutant TP53 and/or Complex Karyotype in Patients with Untreated Acute Myeloid Leukemia ≥Age 60 Years
Source: Cancer Res Commun. 2025 Jul 14;5(7):1129–39. doi: 10.1158/2767-9764.CRC-25-0091 (PMC12257073; doi:10.1158/2767-9764.CRC-25-0091)
Supplement: Supplementary Table S4 — All-Cause Adverse Events [file crc-25-0091_supplementary_table_s4_suppst4.docx]

**Supplementary Table S4. All-Cause Adverse Events**

| **Toxicity Category** | **Adverse Event** | **Group 1 Grade 1 2** | **Group 1 Grade 3+** | **Group 1 All** | **Group 2 Grade 1 2** | **Group 2 Grade 3+** | **Group 2 All** |
| --- | --- | --- | --- | --- | --- | --- | --- |
| Blood and lymphatic system disorders | Neutropenia | 2 (13.3) | 5 (33.3) | 7 (46.7) | 1 (8.3) | 6 (50) | 7 (58.3) |
|  | Thrombocytopenia | 1 (6.7) | 5 (33.3) | 6 (40) | 1 (8.3) | 5 (41.7) | 6 (50) |
|  | Anaemia | 1 (6.7) | 3 (20) | 4 (26.7) | 0 (0) | 3 (25) | 3 (25) |
|  | Febrile neutropenia | 0 (0) | 4 (26.7) | 4 (26.7) | 1 (8.3) | 6 (50) | 7 (58.3) |
|  | Leukopenia | 0 (0) | 4 (26.7) | 4 (26.7) | 2 (16.7) | 6 (50) | 8 (66.7) |
|  | Lymphadenopathy | 0 (0) | 0 (0) | 0 (0) | 1 (8.3) | 0 (0) | 1 (8.3) |
| Cardiac disorders | Cardiac arrest | 0 (0) | 1 (6.7) | 1 (6.7) | 0 (0) | 0 (0) | 0 (0) |
|  | Palpitations | 1 (6.7) | 0 (0) | 1 (6.7) | 0 (0) | 0 (0) | 0 (0) |
|  | Sinus tachycardia | 1 (6.7) | 0 (0) | 1 (6.7) | 0 (0) | 0 (0) | 0 (0) |
|  | Supraventricular tachycardia | 0 (0) | 1 (6.7) | 1 (6.7) | 0 (0) | 0 (0) | 0 (0) |
|  | Tachycardia | 0 (0) | 1 (6.7) | 1 (6.7) | 0 (0) | 0 (0) | 0 (0) |
|  | Atrial flutter | 0 (0) | 0 (0) | 0 (0) | 1 (8.3) | 0 (0) | 1 (8.3) |
|  | Pericarditis | 0 (0) | 0 (0) | 0 (0) | 0 (0) | 1 (8.3) | 1 (8.3) |
| Ear and labyrinth disorders | Deafness unilateral | 1 (6.7) | 0 (0) | 1 (6.7) | 1 (8.3) | 0 (0) | 1 (8.3) |
|  | Ear discomfort | 1 (6.7) | 0 (0) | 1 (6.7) | 0 (0) | 0 (0) | 0 (0) |
|  | Ear pain | 0 (0) | 0 (0) | 0 (0) | 1 (8.3) | 0 (0) | 1 (8.3) |
| Eye disorders | Vision blurred | 2 (13.3) | 0 (0) | 2 (13.3) | 0 (0) | 0 (0) | 0 (0) |
|  | Scleral haemorrhage | 1 (6.7) | 0 (0) | 1 (6.7) | 1 (8.3) | 0 (0) | 1 (8.3) |
|  | Diplopia | 0 (0) | 0 (0) | 0 (0) | 1 (8.3) | 0 (0) | 1 (8.3) |
|  | Photophobia | 0 (0) | 0 (0) | 0 (0) | 1 (8.3) | 0 (0) | 1 (8.3) |
| Gastrointestinal disorders | Nausea | 9 (60) | 0 (0) | 9 (60) | 9 (75) | 0 (0) | 9 (75) |
|  | Diarrhoea | 7 (46.7) | 0 (0) | 7 (46.7) | 6 (50) | 0 (0) | 6 (50) |
|  | Constipation | 6 (40) | 0 (0) | 6 (40) | 6 (50) | 0 (0) | 6 (50) |
|  | Stomatitis | 3 (20) | 1 (6.7) | 4 (26.7) | 1 (8.3) | 0 (0) | 1 (8.3) |
|  | Vomiting | 4 (26.7) | 0 (0) | 4 (26.7) | 3 (25) | 0 (0) | 3 (25) |
|  | Abdominal pain | 2 (13.3) | 0 (0) | 2 (13.3) | 4 (33.3) | 0 (0) | 4 (33.3) |
|  | Gingival bleeding | 2 (13.3) | 0 (0) | 2 (13.3) | 0 (0) | 0 (0) | 0 (0) |
|  | Abdominal distension | 1 (6.7) | 0 (0) | 1 (6.7) | 2 (16.7) | 0 (0) | 2 (16.7) |
|  | Anal fistula | 1 (6.7) | 0 (0) | 1 (6.7) | 0 (0) | 0 (0) | 0 (0) |
|  | Colitis | 0 (0) | 1 (6.7) | 1 (6.7) | 0 (0) | 0 (0) | 0 (0) |
|  | Flatulence | 1 (6.7) | 0 (0) | 1 (6.7) | 1 (8.3) | 0 (0) | 1 (8.3) |
|  | Gastric ulcer | 0 (0) | 1 (6.7) | 1 (6.7) | 0 (0) | 0 (0) | 0 (0) |
|  | Gastrointestinal haemorrhage | 0 (0) | 1 (6.7) | 1 (6.7) | 0 (0) | 0 (0) | 0 (0) |
|  | Gastrooesophageal reflux disease | 1 (6.7) | 0 (0) | 1 (6.7) | 1 (8.3) | 0 (0) | 1 (8.3) |
|  | Haemorrhoids | 1 (6.7) | 0 (0) | 1 (6.7) | 2 (16.7) | 0 (0) | 2 (16.7) |
|  | Lip dry | 1 (6.7) | 0 (0) | 1 (6.7) | 0 (0) | 0 (0) | 0 (0) |
|  | Oral pain | 1 (6.7) | 0 (0) | 1 (6.7) | 0 (0) | 0 (0) | 0 (0) |
|  | Proctalgia | 1 (6.7) | 0 (0) | 1 (6.7) | 2 (16.7) | 0 (0) | 2 (16.7) |
|  | Anal fissure | 0 (0) | 0 (0) | 0 (0) | 0 (0) | 1 (8.3) | 1 (8.3) |
|  | Angina bullosa haemorrhagica | 0 (0) | 0 (0) | 0 (0) | 2 (16.7) | 0 (0) | 2 (16.7) |
|  | Aphthous ulcer | 0 (0) | 0 (0) | 0 (0) | 1 (8.3) | 0 (0) | 1 (8.3) |
|  | Dry mouth | 0 (0) | 0 (0) | 0 (0) | 2 (16.7) | 0 (0) | 2 (16.7) |
|  | Dyspepsia | 0 (0) | 0 (0) | 0 (0) | 1 (8.3) | 0 (0) | 1 (8.3) |
|  | Dysphagia | 0 (0) | 0 (0) | 0 (0) | 1 (8.3) | 0 (0) | 1 (8.3) |
|  | Enterocolitis | 0 (0) | 0 (0) | 0 (0) | 0 (0) | 1 (8.3) | 1 (8.3) |
|  | Gingival pain | 0 (0) | 0 (0) | 0 (0) | 1 (8.3) | 0 (0) | 1 (8.3) |
|  | Gingival swelling | 0 (0) | 0 (0) | 0 (0) | 1 (8.3) | 0 (0) | 1 (8.3) |
|  | Glossodynia | 0 (0) | 0 (0) | 0 (0) | 1 (8.3) | 0 (0) | 1 (8.3) |
|  | Loose tooth | 0 (0) | 0 (0) | 0 (0) | 0 (0) | 1 (8.3) | 1 (8.3) |
|  | Mouth haemorrhage | 0 (0) | 0 (0) | 0 (0) | 1 (8.3) | 0 (0) | 1 (8.3) |
|  | Mouth ulceration | 0 (0) | 0 (0) | 0 (0) | 1 (8.3) | 0 (0) | 1 (8.3) |
|  | Oral disorder | 0 (0) | 0 (0) | 0 (0) | 1 (8.3) | 0 (0) | 1 (8.3) |
|  | Rectal fissure | 0 (0) | 0 (0) | 0 (0) | 1 (8.3) | 0 (0) | 1 (8.3) |
|  | Salivary hypersecretion | 0 (0) | 0 (0) | 0 (0) | 1 (8.3) | 0 (0) | 1 (8.3) |
|  | Upper gastrointestinal haemorrhage | 0 (0) | 0 (0) | 0 (0) | 0 (0) | 1 (8.3) | 1 (8.3) |
| General disorders and administration site conditions | Oedema peripheral | 5 (33.3) | 0 (0) | 5 (33.3) | 4 (33.3) | 0 (0) | 4 (33.3) |
|  | Chills | 4 (26.7) | 0 (0) | 4 (26.7) | 2 (16.7) | 0 (0) | 2 (16.7) |
|  | Fatigue | 3 (20) | 1 (6.7) | 4 (26.7) | 3 (25) | 0 (0) | 3 (25) |
|  | Asthenia | 2 (13.3) | 0 (0) | 2 (13.3) | 1 (8.3) | 0 (0) | 1 (8.3) |
|  | Oedema | 2 (13.3) | 0 (0) | 2 (13.3) | 0 (0) | 0 (0) | 0 (0) |
|  | Pyrexia | 2 (13.3) | 0 (0) | 2 (13.3) | 0 (0) | 0 (0) | 0 (0) |
|  | Cyst | 1 (6.7) | 0 (0) | 1 (6.7) | 0 (0) | 0 (0) | 0 (0) |
|  | Gait disturbance | 1 (6.7) | 0 (0) | 1 (6.7) | 0 (0) | 0 (0) | 0 (0) |
|  | Hernia pain | 1 (6.7) | 0 (0) | 1 (6.7) | 0 (0) | 0 (0) | 0 (0) |
|  | Localised oedema | 1 (6.7) | 0 (0) | 1 (6.7) | 0 (0) | 0 (0) | 0 (0) |
|  | Mucosal inflammation | 0 (0) | 1 (6.7) | 1 (6.7) | 0 (0) | 0 (0) | 0 (0) |
|  | Non-cardiac chest pain | 1 (6.7) | 0 (0) | 1 (6.7) | 2 (16.7) | 0 (0) | 2 (16.7) |
|  | Catheter site bruise | 0 (0) | 0 (0) | 0 (0) | 1 (8.3) | 0 (0) | 1 (8.3) |
|  | Catheter site inflammation | 0 (0) | 0 (0) | 0 (0) | 1 (8.3) | 0 (0) | 1 (8.3) |
|  | Catheter site pain | 0 (0) | 0 (0) | 0 (0) | 2 (16.7) | 0 (0) | 2 (16.7) |
|  | Disease progression | 0 (0) | 0 (0) | 0 (0) | 0 (0) | 1 (8.3) | 1 (8.3) |
|  | Facial pain | 0 (0) | 0 (0) | 0 (0) | 1 (8.3) | 0 (0) | 1 (8.3) |
|  | Injection site erythema | 0 (0) | 0 (0) | 0 (0) | 1 (8.3) | 0 (0) | 1 (8.3) |
|  | Multiple organ dysfunction syndrome | 0 (0) | 0 (0) | 0 (0) | 0 (0) | 1 (8.3) | 1 (8.3) |
|  | Nodule | 0 (0) | 0 (0) | 0 (0) | 1 (8.3) | 0 (0) | 1 (8.3) |
|  | Pain | 0 (0) | 0 (0) | 0 (0) | 1 (8.3) | 0 (0) | 1 (8.3) |
| Hepatobiliary disorders | Ocular icterus | 1 (6.7) | 0 (0) | 1 (6.7) | 0 (0) | 0 (0) | 0 (0) |
|  | Hepatic cyst | 0 (0) | 0 (0) | 0 (0) | 1 (8.3) | 0 (0) | 1 (8.3) |
| Infections and infestations | Candida infection | 2 (13.3) | 0 (0) | 2 (13.3) | 2 (16.7) | 0 (0) | 2 (16.7) |
|  | Bacteraemia | 0 (0) | 1 (6.7) | 1 (6.7) | 2 (16.7) | 0 (0) | 2 (16.7) |
|  | Enterocolitis infectious | 1 (6.7) | 0 (0) | 1 (6.7) | 0 (0) | 0 (0) | 0 (0) |
|  | Localised infection | 1 (6.7) | 0 (0) | 1 (6.7) | 0 (0) | 0 (0) | 0 (0) |
|  | Oral candidiasis | 1 (6.7) | 0 (0) | 1 (6.7) | 0 (0) | 0 (0) | 0 (0) |
|  | Pneumonia | 0 (0) | 1 (6.7) | 1 (6.7) | 0 (0) | 1 (8.3) | 1 (8.3) |
|  | Pneumonia fungal | 0 (0) | 1 (6.7) | 1 (6.7) | 0 (0) | 1 (8.3) | 1 (8.3) |
|  | Rash pustular | 1 (6.7) | 0 (0) | 1 (6.7) | 0 (0) | 0 (0) | 0 (0) |
|  | Wound infection | 0 (0) | 1 (6.7) | 1 (6.7) | 0 (0) | 0 (0) | 0 (0) |
|  | Bronchitis | 0 (0) | 0 (0) | 0 (0) | 1 (8.3) | 0 (0) | 1 (8.3) |
|  | Cellulitis | 0 (0) | 0 (0) | 0 (0) | 0 (0) | 1 (8.3) | 1 (8.3) |
|  | Chorioretinitis | 0 (0) | 0 (0) | 0 (0) | 0 (0) | 1 (8.3) | 1 (8.3) |
|  | Corona virus infection | 0 (0) | 0 (0) | 0 (0) | 0 (0) | 1 (8.3) | 1 (8.3) |
|  | Enterococcal infection | 0 (0) | 0 (0) | 0 (0) | 1 (8.3) | 0 (0) | 1 (8.3) |
|  | Hordeolum | 0 (0) | 0 (0) | 0 (0) | 1 (8.3) | 0 (0) | 1 (8.3) |
|  | Lung infection | 0 (0) | 0 (0) | 0 (0) | 0 (0) | 1 (8.3) | 1 (8.3) |
|  | Mycobacterium abscessus infection | 0 (0) | 0 (0) | 0 (0) | 0 (0) | 1 (8.3) | 1 (8.3) |
|  | Perirectal abscess | 0 (0) | 0 (0) | 0 (0) | 0 (0) | 1 (8.3) | 1 (8.3) |
|  | Rectal abscess | 0 (0) | 0 (0) | 0 (0) | 1 (8.3) | 0 (0) | 1 (8.3) |
|  | Sepsis | 0 (0) | 0 (0) | 0 (0) | 0 (0) | 2 (16.7) | 2 (16.7) |
|  | Septic shock | 0 (0) | 0 (0) | 0 (0) | 0 (0) | 1 (8.3) | 1 (8.3) |
|  | Skin infection | 0 (0) | 0 (0) | 0 (0) | 1 (8.3) | 0 (0) | 1 (8.3) |
|  | Staphylococcal infection | 0 (0) | 0 (0) | 0 (0) | 1 (8.3) | 0 (0) | 1 (8.3) |
| Injury, poisoning and procedural complications | Fall | 1 (6.7) | 0 (0) | 1 (6.7) | 0 (0) | 0 (0) | 0 (0) |
|  | Laceration | 1 (6.7) | 0 (0) | 1 (6.7) | 0 (0) | 0 (0) | 0 (0) |
|  | Seroma | 1 (6.7) | 0 (0) | 1 (6.7) | 0 (0) | 0 (0) | 0 (0) |
|  | Transfusion reaction | 1 (6.7) | 0 (0) | 1 (6.7) | 0 (0) | 0 (0) | 0 (0) |
|  | Vascular access complication | 0 (0) | 1 (6.7) | 1 (6.7) | 0 (0) | 0 (0) | 0 (0) |
|  | Head injury | 0 (0) | 0 (0) | 0 (0) | 1 (8.3) | 0 (0) | 1 (8.3) |
|  | Limb injury | 0 (0) | 0 (0) | 0 (0) | 1 (8.3) | 0 (0) | 1 (8.3) |
|  | Procedural pain | 0 (0) | 0 (0) | 0 (0) | 2 (16.7) | 0 (0) | 2 (16.7) |
|  | Scratch | 0 (0) | 0 (0) | 0 (0) | 1 (8.3) | 0 (0) | 1 (8.3) |
|  | Skin injury | 0 (0) | 0 (0) | 0 (0) | 1 (8.3) | 0 (0) | 1 (8.3) |
| Investigations | Lymphocyte count decreased | 3 (20) | 0 (0) | 3 (20) | 0 (0) | 5 (41.7) | 5 (41.7) |
|  | Blood lactate dehydrogenase increased | 2 (13.3) | 0 (0) | 2 (13.3) | 0 (0) | 0 (0) | 0 (0) |
|  | International normalised ratio increased | 2 (13.3) | 0 (0) | 2 (13.3) | 2 (16.7) | 0 (0) | 2 (16.7) |
|  | Weight decreased | 2 (13.3) | 0 (0) | 2 (13.3) | 0 (0) | 0 (0) | 0 (0) |
|  | Activated partial thromboplastin time prolonged | 1 (6.7) | 0 (0) | 1 (6.7) | 2 (16.7) | 0 (0) | 2 (16.7) |
|  | Alanine aminotransferase increased | 1 (6.7) | 0 (0) | 1 (6.7) | 2 (16.7) | 0 (0) | 2 (16.7) |
|  | Aspartate aminotransferase increased | 1 (6.7) | 0 (0) | 1 (6.7) | 3 (25) | 0 (0) | 3 (25) |
|  | Blood bilirubin increased | 0 (0) | 1 (6.7) | 1 (6.7) | 2 (16.7) | 0 (0) | 2 (16.7) |
|  | Blood creatine increased | 1 (6.7) | 0 (0) | 1 (6.7) | 0 (0) | 0 (0) | 0 (0) |
|  | Lymphocyte count increased | 1 (6.7) | 0 (0) | 1 (6.7) | 1 (8.3) | 0 (0) | 1 (8.3) |
|  | Bilirubin conjugated increased | 0 (0) | 0 (0) | 0 (0) | 1 (8.3) | 0 (0) | 1 (8.3) |
|  | Blood alkaline phosphatase increased | 0 (0) | 0 (0) | 0 (0) | 2 (16.7) | 0 (0) | 2 (16.7) |
|  | Blood bicarbonate decreased | 0 (0) | 0 (0) | 0 (0) | 1 (8.3) | 0 (0) | 1 (8.3) |
|  | Blood creatinine increased | 0 (0) | 0 (0) | 0 (0) | 1 (8.3) | 1 (8.3) | 2 (16.7) |
|  | Blood fibrinogen decreased | 0 (0) | 0 (0) | 0 (0) | 1 (8.3) | 0 (0) | 1 (8.3) |
|  | Cardiac murmur | 0 (0) | 0 (0) | 0 (0) | 1 (8.3) | 0 (0) | 1 (8.3) |
|  | Troponin I increased | 0 (0) | 0 (0) | 0 (0) | 1 (8.3) | 0 (0) | 1 (8.3) |
|  | Urine output decreased | 0 (0) | 0 (0) | 0 (0) | 0 (0) | 1 (8.3) | 1 (8.3) |
| Metabolism and nutrition disorders | Hypoalbuminaemia | 5 (33.3) | 0 (0) | 5 (33.3) | 4 (33.3) | 1 (8.3) | 5 (41.7) |
|  | Hypokalaemia | 5 (33.3) | 0 (0) | 5 (33.3) | 3 (25) | 1 (8.3) | 4 (33.3) |
|  | Hypomagnesaemia | 5 (33.3) | 0 (0) | 5 (33.3) | 1 (8.3) | 0 (0) | 1 (8.3) |
|  | Hyponatraemia | 3 (20) | 1 (6.7) | 4 (26.7) | 3 (25) | 0 (0) | 3 (25) |
|  | Hyperphosphataemia | 2 (13.3) | 0 (0) | 2 (13.3) | 1 (8.3) | 0 (0) | 1 (8.3) |
|  | Decreased appetite | 1 (6.7) | 0 (0) | 1 (6.7) | 4 (33.3) | 1 (8.3) | 5 (41.7) |
|  | Dehydration | 1 (6.7) | 0 (0) | 1 (6.7) | 0 (0) | 0 (0) | 0 (0) |
|  | Diabetes mellitus | 0 (0) | 1 (6.7) | 1 (6.7) | 0 (0) | 0 (0) | 0 (0) |
|  | Hyperglycaemia | 1 (6.7) | 0 (0) | 1 (6.7) | 1 (8.3) | 0 (0) | 1 (8.3) |
|  | Hypophagia | 1 (6.7) | 0 (0) | 1 (6.7) | 0 (0) | 0 (0) | 0 (0) |
|  | Hypophosphataemia | 1 (6.7) | 0 (0) | 1 (6.7) | 1 (8.3) | 0 (0) | 1 (8.3) |
|  | Hyperkalaemia | 0 (0) | 0 (0) | 0 (0) | 1 (8.3) | 1 (8.3) | 2 (16.7) |
|  | Hypertriglyceridaemia | 0 (0) | 0 (0) | 0 (0) | 1 (8.3) | 1 (8.3) | 2 (16.7) |
|  | Hypocalcaemia | 0 (0) | 0 (0) | 0 (0) | 2 (16.7) | 0 (0) | 2 (16.7) |
|  | Metabolic acidosis | 0 (0) | 0 (0) | 0 (0) | 0 (0) | 1 (8.3) | 1 (8.3) |
| Musculoskeletal and connective tissue disorders | Arthralgia | 2 (13.3) | 0 (0) | 2 (13.3) | 2 (16.7) | 0 (0) | 2 (16.7) |
|  | Bone pain | 2 (13.3) | 0 (0) | 2 (13.3) | 0 (0) | 0 (0) | 0 (0) |
|  | Musculoskeletal pain | 2 (13.3) | 0 (0) | 2 (13.3) | 2 (16.7) | 0 (0) | 2 (16.7) |
|  | Neck pain | 2 (13.3) | 0 (0) | 2 (13.3) | 0 (0) | 1 (8.3) | 1 (8.3) |
|  | Arthritis | 1 (6.7) | 0 (0) | 1 (6.7) | 0 (0) | 0 (0) | 0 (0) |
|  | Kyphosis | 1 (6.7) | 0 (0) | 1 (6.7) | 0 (0) | 0 (0) | 0 (0) |
|  | Musculoskeletal chest pain | 1 (6.7) | 0 (0) | 1 (6.7) | 0 (0) | 0 (0) | 0 (0) |
|  | Myalgia | 1 (6.7) | 0 (0) | 1 (6.7) | 0 (0) | 0 (0) | 0 (0) |
|  | Pain in extremity | 1 (6.7) | 0 (0) | 1 (6.7) | 3 (25) | 0 (0) | 3 (25) |
|  | Back pain | 0 (0) | 0 (0) | 0 (0) | 2 (16.7) | 1 (8.3) | 3 (25) |
|  | Flank pain | 0 (0) | 0 (0) | 0 (0) | 1 (8.3) | 0 (0) | 1 (8.3) |
|  | Joint effusion | 0 (0) | 0 (0) | 0 (0) | 0 (0) | 1 (8.3) | 1 (8.3) |
|  | Muscular weakness | 0 (0) | 0 (0) | 0 (0) | 1 (8.3) | 0 (0) | 1 (8.3) |
| Neoplasms benign, malignant and unspecified (incl cysts and polyps) | Acute myeloid leukaemia | 1 (6.7) | 0 (0) | 1 (6.7) | 0 (0) | 0 (0) | 0 (0) |
|  | Seborrhoeic keratosis | 1 (6.7) | 0 (0) | 1 (6.7) | 0 (0) | 0 (0) | 0 (0) |
|  | Cancer pain | 0 (0) | 0 (0) | 0 (0) | 1 (8.3) | 0 (0) | 1 (8.3) |
|  | Spinal cord neoplasm | 0 (0) | 0 (0) | 0 (0) | 0 (0) | 1 (8.3) | 1 (8.3) |
| Nervous system disorders | Dizziness | 5 (33.3) | 0 (0) | 5 (33.3) | 5 (41.7) | 0 (0) | 5 (41.7) |
|  | Headache | 3 (20) | 0 (0) | 3 (20) | 3 (25) | 2 (16.7) | 5 (41.7) |
|  | Dysgeusia | 2 (13.3) | 0 (0) | 2 (13.3) | 1 (8.3) | 0 (0) | 1 (8.3) |
|  | Presyncope | 1 (6.7) | 1 (6.7) | 2 (13.3) | 1 (8.3) | 0 (0) | 1 (8.3) |
|  | Tremor | 2 (13.3) | 0 (0) | 2 (13.3) | 3 (25) | 0 (0) | 3 (25) |
|  | Cerebrovascular accident | 0 (0) | 1 (6.7) | 1 (6.7) | 0 (0) | 0 (0) | 0 (0) |
|  | Cognitive disorder | 1 (6.7) | 0 (0) | 1 (6.7) | 0 (0) | 0 (0) | 0 (0) |
|  | Paraesthesia | 1 (6.7) | 0 (0) | 1 (6.7) | 0 (0) | 0 (0) | 0 (0) |
|  | Peripheral sensory neuropathy | 1 (6.7) | 0 (0) | 1 (6.7) | 0 (0) | 0 (0) | 0 (0) |
|  | Syncope | 0 (0) | 1 (6.7) | 1 (6.7) | 0 (0) | 3 (25) | 3 (25) |
|  | Depressed level of consciousness | 0 (0) | 0 (0) | 0 (0) | 1 (8.3) | 0 (0) | 1 (8.3) |
|  | Encephalopathy | 0 (0) | 0 (0) | 0 (0) | 3 (25) | 1 (8.3) | 4 (33.3) |
|  | Haemorrhage intracranial | 0 (0) | 0 (0) | 0 (0) | 0 (0) | 1 (8.3) | 1 (8.3) |
|  | Lethargy | 0 (0) | 0 (0) | 0 (0) | 2 (16.7) | 0 (0) | 2 (16.7) |
| Psychiatric disorders | Insomnia | 6 (40) | 0 (0) | 6 (40) | 3 (25) | 0 (0) | 3 (25) |
|  | Anxiety | 1 (6.7) | 0 (0) | 1 (6.7) | 0 (0) | 0 (0) | 0 (0) |
|  | Delirium | 1 (6.7) | 0 (0) | 1 (6.7) | 1 (8.3) | 0 (0) | 1 (8.3) |
|  | Depression | 1 (6.7) | 0 (0) | 1 (6.7) | 1 (8.3) | 0 (0) | 1 (8.3) |
|  | Confusional state | 0 (0) | 0 (0) | 0 (0) | 1 (8.3) | 1 (8.3) | 2 (16.7) |
|  | Hallucination | 0 (0) | 0 (0) | 0 (0) | 2 (16.7) | 0 (0) | 2 (16.7) |
| Renal and urinary disorders | Haematuria | 2 (13.3) | 0 (0) | 2 (13.3) | 2 (16.7) | 0 (0) | 2 (16.7) |
|  | Chronic kidney disease | 1 (6.7) | 0 (0) | 1 (6.7) | 0 (0) | 0 (0) | 0 (0) |
|  | Renal cyst | 1 (6.7) | 0 (0) | 1 (6.7) | 0 (0) | 0 (0) | 0 (0) |
|  | Urinary incontinence | 1 (6.7) | 0 (0) | 1 (6.7) | 3 (25) | 0 (0) | 3 (25) |
|  | Urinary retention | 1 (6.7) | 0 (0) | 1 (6.7) | 2 (16.7) | 0 (0) | 2 (16.7) |
|  | Acute kidney injury | 0 (0) | 0 (0) | 0 (0) | 0 (0) | 3 (25) | 3 (25) |
|  | Chromaturia | 0 (0) | 0 (0) | 0 (0) | 2 (16.7) | 0 (0) | 2 (16.7) |
|  | Glycosuria | 0 (0) | 0 (0) | 0 (0) | 1 (8.3) | 0 (0) | 1 (8.3) |
|  | Nephrolithiasis | 0 (0) | 0 (0) | 0 (0) | 1 (8.3) | 0 (0) | 1 (8.3) |
|  | Pollakiuria | 0 (0) | 0 (0) | 0 (0) | 2 (16.7) | 0 (0) | 2 (16.7) |
|  | Proteinuria | 0 (0) | 0 (0) | 0 (0) | 2 (16.7) | 0 (0) | 2 (16.7) |
| Reproductive system and breast disorders | Pelvic pain | 0 (0) | 0 (0) | 0 (0) | 1 (8.3) | 0 (0) | 1 (8.3) |
|  | Prostatomegaly | 0 (0) | 0 (0) | 0 (0) | 1 (8.3) | 0 (0) | 1 (8.3) |
| Respiratory, thoracic and mediastinal disorders | Dyspnoea | 6 (40) | 0 (0) | 6 (40) | 5 (41.7) | 1 (8.3) | 6 (50) |
|  | Cough | 3 (20) | 0 (0) | 3 (20) | 5 (41.7) | 0 (0) | 5 (41.7) |
|  | Epistaxis | 2 (13.3) | 0 (0) | 2 (13.3) | 1 (8.3) | 0 (0) | 1 (8.3) |
|  | Wheezing | 2 (13.3) | 0 (0) | 2 (13.3) | 1 (8.3) | 1 (8.3) | 2 (16.7) |
|  | Hypoxia | 0 (0) | 1 (6.7) | 1 (6.7) | 0 (0) | 1 (8.3) | 1 (8.3) |
|  | Oropharyngeal pain | 1 (6.7) | 0 (0) | 1 (6.7) | 3 (25) | 0 (0) | 3 (25) |
|  | Respiratory distress | 0 (0) | 1 (6.7) | 1 (6.7) | 0 (0) | 0 (0) | 0 (0) |
|  | Respiratory failure | 0 (0) | 1 (6.7) | 1 (6.7) | 0 (0) | 2 (16.7) | 2 (16.7) |
|  | Rhinorrhoea | 1 (6.7) | 0 (0) | 1 (6.7) | 1 (8.3) | 0 (0) | 1 (8.3) |
|  | Tachypnoea | 1 (6.7) | 0 (0) | 1 (6.7) | 0 (0) | 0 (0) | 0 (0) |
|  | Acute respiratory distress syndrome | 0 (0) | 0 (0) | 0 (0) | 0 (0) | 1 (8.3) | 1 (8.3) |
|  | Atelectasis | 0 (0) | 0 (0) | 0 (0) | 1 (8.3) | 0 (0) | 1 (8.3) |
|  | Nasal congestion | 0 (0) | 0 (0) | 0 (0) | 2 (16.7) | 0 (0) | 2 (16.7) |
|  | Pleural effusion | 0 (0) | 0 (0) | 0 (0) | 3 (25) | 0 (0) | 3 (25) |
|  | Productive cough | 0 (0) | 0 (0) | 0 (0) | 2 (16.7) | 0 (0) | 2 (16.7) |
|  | Rales | 0 (0) | 0 (0) | 0 (0) | 1 (8.3) | 0 (0) | 1 (8.3) |
|  | Sneezing | 0 (0) | 0 (0) | 0 (0) | 1 (8.3) | 0 (0) | 1 (8.3) |
| Skin and subcutaneous tissue disorders | Alopecia | 2 (13.3) | 0 (0) | 2 (13.3) | 1 (8.3) | 0 (0) | 1 (8.3) |
|  | Dry skin | 1 (6.7) | 0 (0) | 1 (6.7) | 0 (0) | 0 (0) | 0 (0) |
|  | Ecchymosis | 1 (6.7) | 0 (0) | 1 (6.7) | 4 (33.3) | 0 (0) | 4 (33.3) |
|  | Erythema | 1 (6.7) | 0 (0) | 1 (6.7) | 0 (0) | 0 (0) | 0 (0) |
|  | Nail discolouration | 1 (6.7) | 0 (0) | 1 (6.7) | 0 (0) | 0 (0) | 0 (0) |
|  | Photosensitivity reaction | 1 (6.7) | 0 (0) | 1 (6.7) | 0 (0) | 0 (0) | 0 (0) |
|  | Pruritus | 1 (6.7) | 0 (0) | 1 (6.7) | 0 (0) | 0 (0) | 0 (0) |
|  | Rash | 1 (6.7) | 0 (0) | 1 (6.7) | 1 (8.3) | 0 (0) | 1 (8.3) |
|  | Skin disorder | 1 (6.7) | 0 (0) | 1 (6.7) | 0 (0) | 0 (0) | 0 (0) |
|  | Erythema nodosum | 0 (0) | 0 (0) | 0 (0) | 1 (8.3) | 0 (0) | 1 (8.3) |
|  | Hyperhidrosis | 0 (0) | 0 (0) | 0 (0) | 1 (8.3) | 0 (0) | 1 (8.3) |
|  | Petechiae | 0 (0) | 0 (0) | 0 (0) | 3 (25) | 0 (0) | 3 (25) |
|  | Rash papular | 0 (0) | 0 (0) | 0 (0) | 1 (8.3) | 0 (0) | 1 (8.3) |
|  | Scab | 0 (0) | 0 (0) | 0 (0) | 1 (8.3) | 0 (0) | 1 (8.3) |
|  | Skin lesion | 0 (0) | 0 (0) | 0 (0) | 1 (8.3) | 0 (0) | 1 (8.3) |
|  | Swelling face | 0 (0) | 0 (0) | 0 (0) | 1 (8.3) | 0 (0) | 1 (8.3) |
| Vascular disorders | Hypotension | 2 (13.3) | 1 (6.7) | 3 (20) | 0 (0) | 1 (8.3) | 1 (8.3) |
|  | Hypertension | 0 (0) | 1 (6.7) | 1 (6.7) | 0 (0) | 1 (8.3) | 1 (8.3) |
|  | Lymphoedema | 0 (0) | 0 (0) | 0 (0) | 1 (8.3) | 0 (0) | 1 (8.3) |
|  | Peripheral venous disease | 0 (0) | 0 (0) | 0 (0) | 1 (8.3) | 0 (0) | 1 (8.3) |
| Missing Categories | COPD exacerbation | 1 (6.7) | 0 (0) | 1 (6.7) | 0 (0) | 0 (0) | 0 (0) |
|  | Death, NOS | 0 (0) | 1 (6.7) | 1 (6.7) | 0 (0) | 0 (0) | 0 (0) |
|  | Hand-Foot Syndrome | 1 (6.7) | 0 (0) | 1 (6.7) | 0 (0) | 0 (0) | 0 (0) |
|  | Hip Pain | 1 (6.7) | 0 (0) | 1 (6.7) | 0 (0) | 0 (0) | 0 (0) |
|  | INR - Increased | 1 (6.7) | 0 (0) | 1 (6.7) | 0 (0) | 0 (0) | 0 (0) |
|  | Retinal Detachment | 1 (6.7) | 0 (0) | 1 (6.7) | 0 (0) | 0 (0) | 0 (0) |
|  | lung fullness | 1 (6.7) | 0 (0) | 1 (6.7) | 0 (0) | 0 (0) | 0 (0) |
|  | Atrial fibrillation with RVR | 0 (0) | 0 (0) | 0 (0) | 1 (8.3) | 0 (0) | 1 (8.3) |
|  | Chest pain (non-cardiac) | 0 (0) | 0 (0) | 0 (0) | 1 (8.3) | 0 (0) | 1 (8.3) |
|  | Diarrhea (intermittent) | 0 (0) | 0 (0) | 0 (0) | 1 (8.3) | 0 (0) | 1 (8.3) |
|  | Hypokalemia (intermittent) | 0 (0) | 0 (0) | 0 (0) | 1 (8.3) | 0 (0) | 1 (8.3) |
|  | Hypotension (intermittent) | 0 (0) | 0 (0) | 0 (0) | 1 (8.3) | 0 (0) | 1 (8.3) |
|  | Inflammation (bilateral knee) | 0 (0) | 0 (0) | 0 (0) | 1 (8.3) | 0 (0) | 1 (8.3) |
|  | Itching (intermittent) | 0 (0) | 0 (0) | 0 (0) | 1 (8.3) | 0 (0) | 1 (8.3) |
|  | Microvascular ischemic disease | 0 (0) | 0 (0) | 0 (0) | 1 (8.3) | 0 (0) | 1 (8.3) |
|  | Pain (leg) | 0 (0) | 0 (0) | 0 (0) | 1 (8.3) | 0 (0) | 1 (8.3) |
|  | Steroid-induced hyperglycemia | 0 (0) | 0 (0) | 0 (0) | 1 (8.3) | 0 (0) | 1 (8.3) |
|  | Symptomatic anemia | 0 (0) | 0 (0) | 0 (0) | 0 (0) | 1 (8.3) | 1 (8.3) |
|  | neuropathy (BLE) | 0 (0) | 0 (0) | 0 (0) | 1 (8.3) | 0 (0) | 1 (8.3) |
|  | pericarditis (intermittent) | 0 (0) | 0 (0) | 0 (0) | 1 (8.3) | 0 (0) | 1 (8.3) |
