# Supplementary material for: A Phase 1b/2 Study of TP-0903 and Decitabine Targeting Mutant TP53 and/or Complex Karyotype in Patients with Untreated Acute Myeloid Leukemia ≥Age 60 Years
Source: Cancer Res Commun. 2025 Jul 14;5(7):1129–39. doi: 10.1158/2767-9764.CRC-25-0091 (PMC12257073; doi:10.1158/2767-9764.CRC-25-0091)
Supplement: Supplementary Table S5 — Treatment Completion and Reason Off Treatment [file crc-25-0091_supplementary_table_s5_suppst5.docx]

**Supplementary Table S5. Treatment Completion and Reason Off Treatment**

| **All Patients** | **Group 1**  **(N = 15)**  **37 mg** | **Group 2**  **(N = 12)**  **25 mg** | **Overall**  **(N = 27)** |
| --- | --- | --- | --- |
| No. of months on treatment, median (range) | 1.5 (0.1-16.4) | 3.2 (0.2-7.0) | 2.5 (0.1-16.4) |
| No. of cycles of TP-0903 on treatment,  median (range) | 2 (1-16) | 3 (1-6) | 3 (1-16) |
| Off treatment for reasons, n (%)  Adverse event  Treatment failure  Recurrence after a response  Disease progression  Death  Stem cell transplant  Withdrawal consent  Trial discontinued by sponsor  Other | 2 (13.3)  2 (13.3)  2 (13.3)  0 (0)  1 (6.7)  2 (13.3)  4 (26.7)  0 (0)  2 (13.3) | 2 (16.7)  3 (25)  2 (16.7)  2 (16.7)  0 (0)  1 (8.3)  0 (0)  1 (8.3)  1 (8.3) | 4 (14.8)  5 (18.5)  4 (14.8)  2 (7.4)  1 (3.7)  3 (11.1)  4 (14.8)  1 (3.7)  3 (11.1)* |

*one patient went to hospice, one had hip replacement surgery, and one based on the patient's decision.
