# Supplementary material for: A Phase 1b/2 Study of TP-0903 and Decitabine Targeting Mutant TP53 and/or Complex Karyotype in Patients with Untreated Acute Myeloid Leukemia ≥Age 60 Years
Source: Cancer Res Commun. 2025 Jul 14;5(7):1129–39. doi: 10.1158/2767-9764.CRC-25-0091 (PMC12257073; doi:10.1158/2767-9764.CRC-25-0091)
Supplement: Supplementary Table S6 — TP-0903 and metabolite pharmacokinetic parameters on days 1 and 10 of course 1 [file crc-25-0091_supplementary_table_s6_suppst6.docx]

| **Supplementary Table S6. TP-0903 and metabolite pharmacokinetic parameters on days 1 and 10 of course 1** | | | | | | | | | | |
| --- | --- | --- | --- | --- | --- | --- | --- | --- | --- | --- |
|  |  | **Day 1** | | | | **Day 10** | | | | |
| TP-0903 Dose | Compound | Tmax (h) | Cmax (ng/mL) | AUC0-24h (h*ng/mL) | C24h trough (ng/mL) | Pre-treatment trough (ng/mL) | Tmax (h) | Cmax (ng/mL) | AUC0-24h (h*ng/mL) | C24h trough (ng/mL) |
| 25mg | TP-0903 | 2.8 (0.58-23) | 2.4 (0.22-12) | 42 (4.0-181) | 1.9 (0.21-5.6) | 7.1 (3.3-17) | 3.5 (0.13-4.0) | 14 (1.2-44) | 260 (110-690) | 8.1 (0.35-16) |
| (N=11/8) | M2 | 21 (2.1-24) | 0.16 (0.06-0.46) | 2.8 (1.1-7.1) | 0.13 (0.06-0.36) | 0.5 (0.17-2.1) | 3.5 (0.13-23) | 0.54 (0.23-2.9) | 12 (4.4-59) | 0.38 (0.18-2.1) |
|  | M3 | 21 (1.3-24) | 0.9 (0.4-3.3) | 16 (6.2-42) | 0.70 (0.31-1.7) | 2.9 (1.5-8.4) | 3.5 (0.13-8.0) | 3.2 (2.2-13) | 80 (45-260) | 3.0 (1.1-9.4) |
|  | M4 | 3.5 (0.9-23) | 0.3 (0.1-0.7) | 4.1 (2.2-14) | 0.21 (0.08-0.53) | 1.1 (0.6-4.0) | 3.5 (0.1-23) | 1.3 (0.6-6.2) | 29 (22-120) | 1.2 (0.19-4.0) |
|  | M6 | 22 (3.5-24) | 0.043 (0.025-0.253) | 0.54 (0.26-4.6) | 0.05 (0.02-0.20) | 0.14 (0.05-0.67) | 3.5 (0-24) | 0.17 (0.06-0.68) | 3.6 (1.1-14) | 0.13 (0.05-0.60) |
|  | Sum of all species | 2.8 (0.9-23) | 3.5 (0.96-14) | 67 (15-230) | 2.9 (0.87-7.9) | 12 (5.9-30) | 3.5 (0.13-4.0) | 18 (4.9-63) | 400 (180-1100) | 12 (2.0-29) |
| 37 mg | TP-0903 | 2.1 (0.42-7.5) | 3.3 (0.71-23) | 36 (12-340) | 0.70 (0.27-11) | 5.3 (1.0-21) | 3.9 (1.8-7.8) | 13 (1.8-45) | 210 (37-690) | 5.8 (1.2-20) |
| (N=10/9) | M2 | 4.1 (0.98-24) | 0.6 (0.06-2.2) | 7.2 (1.3-17) | 0.19 (0.06-1.8) | 0.7 (0.1-1.9) | 3.5 (1.0-7.6) | 1.4 (0.2-2.6) | 22 (4.2-45) | 0.7 (0.2-1.8) |
|  | M3 | 4.1 (1.8-24) | 2.1 (0.47-7.6) | 31 (8.2-110) | 0.94 (0.1-3.4) | 4.6 (1.1-15) | 4.0 (1.8-8.0) | 7.6 (1.6-24) | 150 (32-410) | 5.5 (1.1-13) |
|  | M4 | 2.1 (1.1-7.5) | 0.8 (0.2-3.7) | 8.6 (3.8-21) | 0.15 (0.06-0.72) | 1.1 (0.3-3.7) | 3.5 (1.8-4.1) | 1.8 (0.7-5.7) | 36 (14-120) | 1.2 (0.4-4.2) |
|  | M6 | 8.3 (4.1-24) | 0.1 (0.04-0.17) | 1.1 (0.63-2.45) | 0.06 (0.03-15) | 0.16 (0.04-0.56) | 4.2 (0-24) | 0.18 (0.04-0.73) | 3.7 (0.86-16) | 0.12 (0.04-0.71) |
|  | Sum of all species | 4.0 (2.0-7.5) | 7.5 (1.5-43) | 86 (26-400) | 2.0 (0.12-5.8) | 11 (3.3-41) | 3.9 (1.8-4.1) | 24 (5.1-71) | 440 (100-1200) | 13 (3.3-37) |
| Number of patients is based on those with AUC_0-24h_ estimates (Day 1/Day 10). Data are the median (range) | | | | | | | | | | |
