# Supplementary material for: A Phase 1b/2 Study of TP-0903 and Decitabine Targeting Mutant TP53 and/or Complex Karyotype in Patients with Untreated Acute Myeloid Leukemia ≥Age 60 Years
Source: Cancer Res Commun. 2025 Jul 14;5(7):1129–39. doi: 10.1158/2767-9764.CRC-25-0091 (PMC12257073; doi:10.1158/2767-9764.CRC-25-0091)
Supplement: Supplementary Table S7 — Research Resource Identifiers (RRID) [file crc-25-0091_supplementary_table_s7_suppst7.docx]

**Supplementary Table S7. Research Resource Identifiers (RRID)**

| **Reagent or Resource** | **Source** | **RRID/Cat#** |
| --- | --- | --- |
| **Spectral Flow** |  |  |
| CD45 clone 2D1 | BD | AB_647361 |
| pH2XA clone HisH2AXS139-1E4 | ThermoFisher | AB_2896984 |
| LIVE/Dead Fixable Blue Dead Cell Stain | ThermoFisher | L23105 |
| Cytofix Fixation Buffer | BD | 554655 |
| Phosflow Perm Buffer III | BD | 558050 |
| Human BD FC block clone Fc1 | BD | AB_2869554 |
| UltraComp eBeads Plus Compensation Beads | ThermoFisher | 01-3333-41 |
| **Software/Instrument (Spectral Flow)** |  |  |
| FAC Express 7 | De novo | SCR_016431 |
| Graphpad Prism |  | SCR_002798 |
| Aurora | Cytek |  |
| **Mutation Analysis** |  |  |
| Covaris ME220 Focused Ultrasonicator | SciCrunch Registry | SCR_019818 |
| Illumina NovaSeq 6000 Sequencing System | SciCrunch Registry | SCR_016387 |
| BWA | SciCrunch Registry | SCR_010910 |
| GATK | SciCrunch Registry | SCR_001876 |
| Mutect2 | SciCrunch Registry | SCR_026692 |
| SnpEff | SciCrunch Registry | SCR_005191 |
| vcfanno | SciCrunch Registry | SCR_024372 |
| dbSNP | SciCrunch Registry | SCR_002338 |
| COSMIC - Catalogue Of Somatic Mutations In Cancer | SciCrunch Registry | SCR_002260 |
| Genome Aggregation Database | SciCrunch Registry | SCR_014964 |
| **Ohio State University Pharmacoanalytical Shared Resource (PhASR)** |  | **RRID: SCR_026701** |
| **Standards for LC-MS/MS** |  |  |
| 2-(5-chloro-2-(4-((4-methylpiperazin-1-yl)methyl)phenylamino)pyrimidin-4-yl)amino-N,N-dimethylbenzene-1-sulfonamide di-tartrate salt (TP-0903) | Tolero/SPD (original CoA from GVK BIO) | Ref #: A-FP-TO5_001, AR210062 |
| M2 | Nucro-technics (CoA Ref from Nanosyn) | Ref #: NSN23144 |
| M3 (SULF-1) | Nucro-technics (CoA Ref from Nanosyn) | Ref #: NSN23087 |
| M4 (SULF-2) | Nucro-technics (CoA Ref from Nanosyn) | Ref #: NSN23088 |
| M6 | Nucro-technics (CoA Ref from Nanosyn) | Ref #: NSN23145 |
| TP-0903-d8 | Nucro-technics (CoA Ref from Nanosyn) | Ref #: NSN23293 |
| M2-d8 | Nucro-technics (CoA Ref from Nanosyn) | Ref #: NSN23290 |
| M3-d8 | Nucro-technics (CoA Ref from Nanosyn) | Ref #: NSN23292 |
| M4-d8 | Nucro-technics (CoA Ref from Nanosyn) | Ref #: NSN23291 |
| M6-d8 | Nucro-technics (CoA Ref from Nanosyn) | Ref #: NSN23296 |
| **Sample Preparation (LC-MS/MS)** |  |  |
| Oasis HLB 30 µM (30 mg) 96-well SPE plate | Waters | Cat #:WAT-058951 |
| Ace EXCEL SuperC18, 3.0 x 75 mm, 5 µm, 90A | MAC-MOD (now a part of Avantor) | Cat #:EXL-1211-7503 |
| Ultrashield Pre-column Filters | MAC-MOD (now a part of Avantor) | Cat #:MMUS1505 |
| **Solvents (LC-MS/MS)** |  |  |
| Ammonium acetate | Sigma-Aldrich | 09689-100G |
| Sodium carbonate | Sigma-Aldrich | 791768-500G |
| Formic acid | ChemProducts | F0441-32 |
| Methanol | Fisher Scientific | A998-4 |
| Acetonitrile | Fisher Scientific | A452-4 |
| **Software/Instruments (LC-MS/MS)** |  |  |
| Vanquish™ Ultra High Pressure Liquid Chromatography System | Thermo Fisher Scientific | RRID:SCR_025713 |
| TSQ Altis™ | Thermo Fisher Scientific | Cat #:TSQ02-10002 |
| Xcalibur™ | Thermo Fisher Scientific | RRID:SCR_014594 |
| Excel | Microsoft | RRID: SCR_016137 |
| Phoenix WinNonlin 8.1 | Certara | RRID: SCR_024504 |
