# Supplementary material for: A Phase 1b/2 Study of TP-0903 and Decitabine Targeting Mutant TP53 and/or Complex Karyotype in Patients with Untreated Acute Myeloid Leukemia ≥Age 60 Years
Source: Cancer Res Commun. 2025 Jul 14;5(7):1129–39. doi: 10.1158/2767-9764.CRC-25-0091 (PMC12257073; doi:10.1158/2767-9764.CRC-25-0091)
Supplement: Supplementary Methods [file crc-25-0091_supplementary_methods_suppsm.docx]

**Supplementary Methods**

*Mutational Analysis*

Genomic DNA was isolated from cryopreserved cells with QIAmp DNA Mini Kit (Qiagen) and fragmented using ME220 Focused-ultrasonicator (Covaris; SCR_019818). Libraries were generated using xGen™ DNA Lib Prep MC UNI kit and target capture was done using xGen AML Cancer Hybridization Panel in conjunction with a 3 gene spike-in pool for *ZRSR2*, *AXL*, and *ASXL2* (Integrated DNA Technologies). DNA library preparations were performed according to the manufacturer’s instructions and sequenced on an Illumina Novaseq (OSUCCC Genomics Shared Resource) (SCR_016387). Sequenced reads were aligned to the GRCh38 genome build using the Burrows-Wheeler Aligner (BWA) (SCR_010910). Picard Tools was used to perform UMI-consensus calling on the aligned reads. The Genome Analysis Toolkit (GATK) (SCR_001876) was used to realign insertions and deletions in the aligned reads and to perform base quality score recalibration for those realigned regions. GATK’s MuTect2 (SCR_026692) was used to perform variant calling. After variant calling, variants were annotated using SnpEff (SCR_005191) and vcfanno (SCR_024372) along with the dbsnp (SCR_002338), COSMIC (SCR_002260), and gnomad variant databases (SCR_014964). The Mucor3 algorithm was used as the baseline for integrative mutation assessment. Visual inspection of all variants was carried out using Integrative Genomics Viewer v.2.8 (Broad Institute).

*LC-MS/MS Analysis of TP-0903 and metabolites*

Sample preparation comprised 200 µL of K2EDTA plasma sample combined with 5 µL of 150 ng/mL internal standard mixture (TP-0903-d8 (NSN23293), M2-d8 (NSN23290), M3-d8 (NSN23292), M4-d8 (NSN23291), and M6-d8 (NSN23296)), 50 µL deionized (DI) water, and 50 µL of methanol in the wells of a 2-mL 96-well plate. Reference standards for TP-0903 (ditartrate salt), metabolites, and internal standards were obtained from Sumitomo Pharma America, Inc. (formerly Sumitomo Pharma Oncology, Inc.). Sample preparation was followed by shaking at 500 rpm for 5 minutes at room temperature and the addition of 1 mL of 0.1M sodium carbonate (791768-500G). After centrifugation at 3095 rcf for 10 minutes, 1 mL of supernatant was loaded onto a conditioned 30 mg HLB SPE plate (conditioning included 1 mL methanol flow through followed by 1 mL water flow through). After samples were loaded and passed through the SPE plate using a vacuum, the plate was washed with 1 mL DI water. Once the water passed through, analyte elution was done by loading 1 mL 2% formic acid in methanol, followed by drying with N_2_ gas heated to 40^o^C. Once dried, they were resuspended with 100 µL reconstitution solution (22% acetonitrile, 22% methanol, 56% DI water, 0.5 mM ammonium acetate, and 0.2% formic acid). The chromatographic method utilized a gradient elution with mobile phase A (MPA; 15% acetonitrile, 15% methanol, 70% DI water, 0.5 mM ammonium acetate, and 0.2% formic acid) and mobile phase B (MPB; 45% acetonitrile, 45% methanol, 10% DI water, 0.5 mM ammonium acetate (09689-100G), and 0.2% formic acid (F0441-32)). The flow rate begins at 0.4 mL/min with 25% MPB, at 2.5 min the flow rate increased to 0.6 mL/min, at 5.5 minutes the %MPB increased to 100%, at 7.01 min the flow rate and %MPB decreased to 0.4 mL/min and 25%, respectively, until the end of the run. This method was run using an ACE Excel SuperC18 (3.0 x 75 mm, 5 µm, 90Å) column (EXL-1211-7503) with a ThermoFisher Scientific Vanquish™ – ThermoFisher Scientific (SCR_025713) TSQ Altis LC-MS/MS system (TSQ02-10002). The analytes and internal standards were evaluated in positive mode with the transitions monitored including M2 488.12->402.125, M2-d8 496.25->402.095, M3 502.2->402.113, M3-d8 510.2->402.113, M4 502.138->416.125, M4-d8 510.188->416.125, M6 518.112->402, M6-d8 526.2->402.113, and TP-0903 516.2->416.125, TP-0903-d8 524.237->416.125. The reportable range was 0.05 – 25 ng/mL, with the exception of M6, which had a reportable range of 0.025 – 10 ng/mL. Three quality control samples (QC Low, QC Medium, and QC High) were monitored during sample analyses to ensure the accuracy of assay quantitation. Additionally, a validation was performed that included the evaluation of selectivity, matrix effects, sensitivity, carry-over, freeze-thaw stability, processed sample stability, recovery, and dilution integrity (20X and 10X). Intra- and inter- day accuracy and precision of the calibrators and quality control samples, which also included the LLOQ for each analyte, was evaluated over 3 days. The quality control samples concentrations were continually monitored by statistical analysis of runs using Westgard rules (1_3s_, 2_2s_, R_4s_, and 10_x_) and adherence to FDA guidance of being within 15% except at the LLOQ of being within 20%. Occasional dilution of samples that were initially above the limit of quantitation was required, with 20X dilution integrity QCs being tested during the analytical runs to confirm that diluted samples were accurately quantitated.

*Analysis of Pharmacokinetics Parameters for TP-0903 and metabolites*

Plasma PK parameters were estimated by noncompartmental analysis using Phoenix WinNonlin 8.1 (Certara) (SCR_024504). Maximum plasma concentration (C_max_), the time to C_max_ (T_max_), the area under the concentration-time curve from time 0 to 24 h (AUC_0–24h_), and steady-state trough levels (concentrations at time 0 on day 10; C_ss_ Min) were estimated from the observed concentration-time data. Plasma was not collected on day 10 from three patients in Group 1 and two patients in Group 2. Patients were excluded from AUC summary statistics if a 24 h sample was not collected or if they received the next day’s TP-0903 dose prior to the collection of the 24 h sample.
