# Supplementary figures and images for: A Phase 1b/2 Study of TP-0903 and Decitabine Targeting Mutant TP53 and/or Complex Karyotype in Patients with Untreated Acute Myeloid Leukemia ≥Age 60 Years
Source: Cancer Res Commun. 2025 Jul 14;5(7):1129–39. doi: 10.1158/2767-9764.CRC-25-0091 (PMC12257073; doi:10.1158/2767-9764.CRC-25-0091)

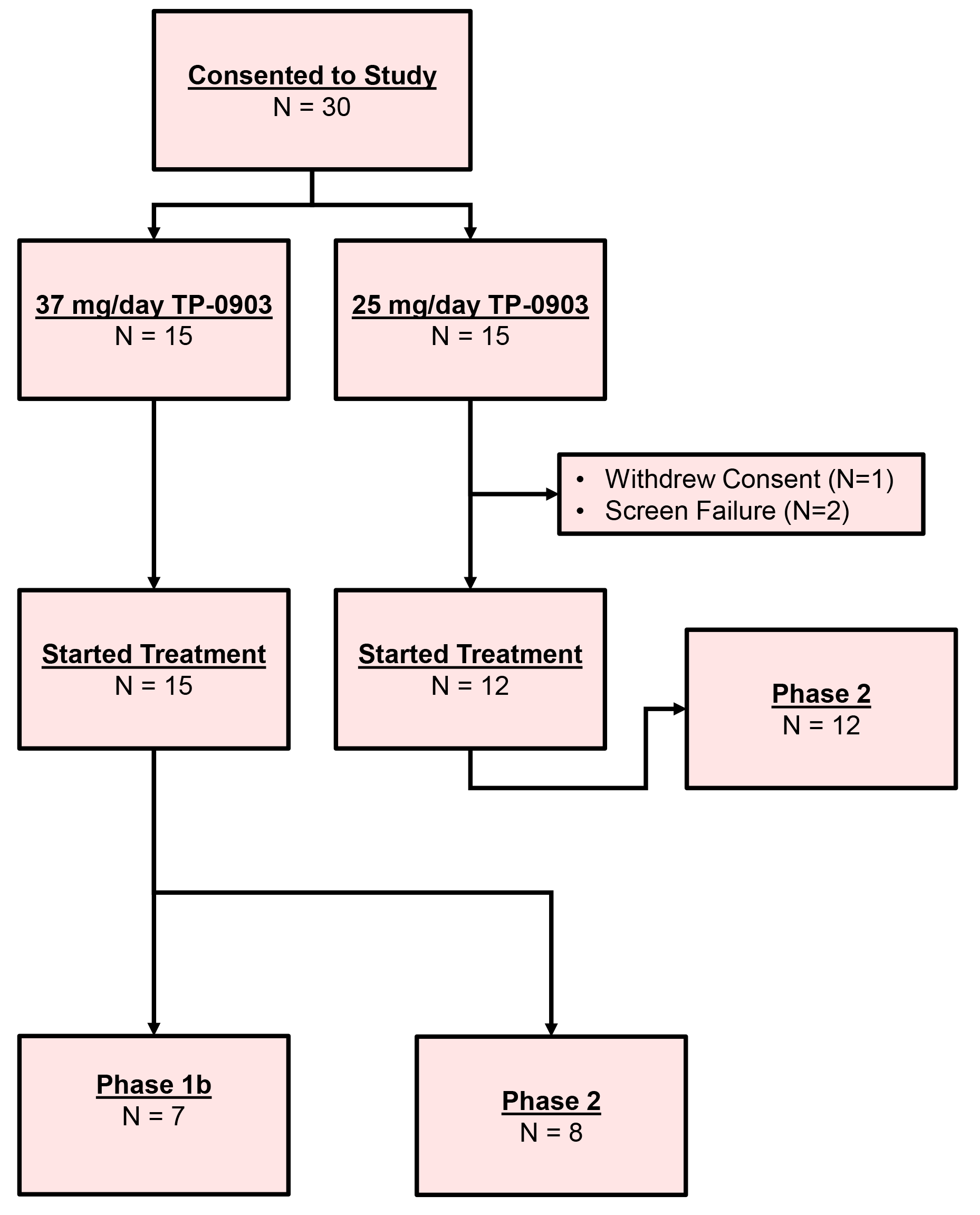

Supplement: Supplementary Figure S1 — Consort diagram [file crc-25-0091_supplementary_figure_s1_suppsf1.png]

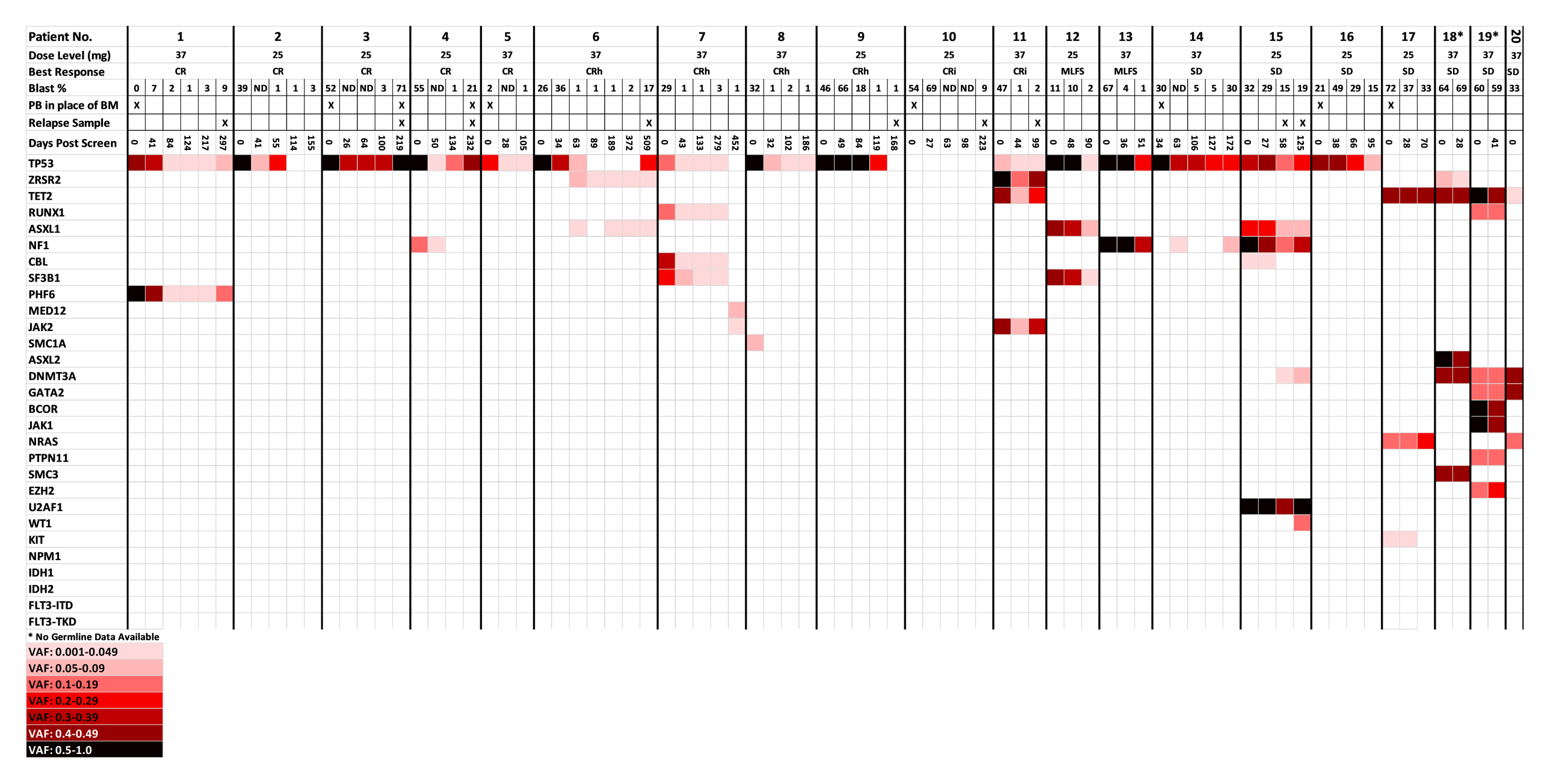

Supplement: Supplementary Figure S2 — Sequencing data. Serial sequencing versus response. [file crc-25-0091_supplementary_figure_s2_suppsf2.png]

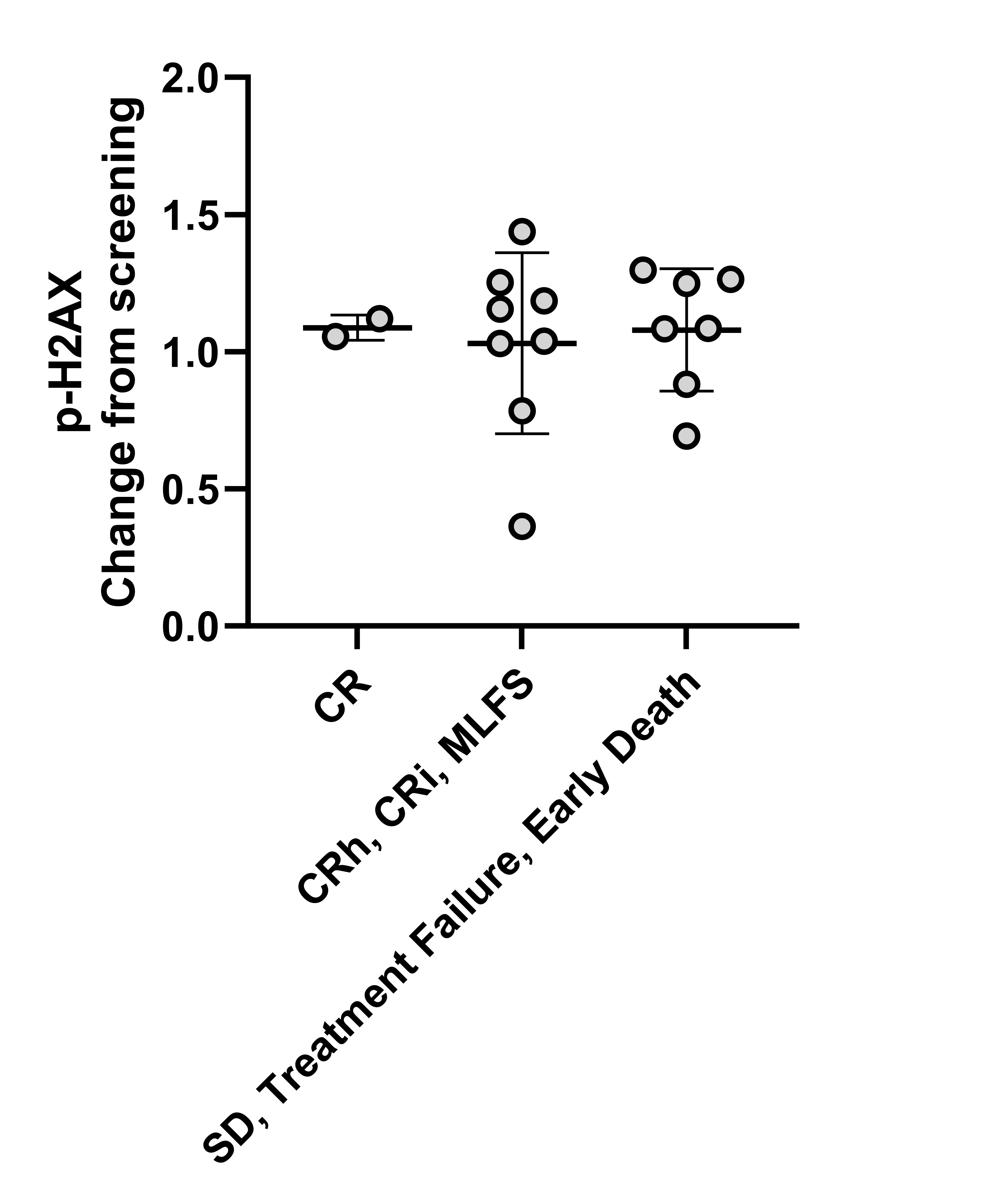

Supplement: Supplementary Figure S4 — p-H2AX as a pharmacodynamic biomarker for TP-0903. Spectral flow cytometry was performed to assess p-H2AX using peripheral blood collected at day 1 (i.e., baseline) and day 10, and change from screening was calculated for patients that achieved CR, patients that achieved CRh, CRi, or MLFS, and non-responders. [file crc-25-0091_supplementary_figure_s4_suppsf4.png]
